# Mitochondria antioxidant protection against cardiovascular dysfunction programmed by early-onset gestational hypoxia

**DOI:** 10.1096/fj.202002705R

**Published:** 2021-05-01

**Authors:** Ana-Mishel Spiroski, Youguo Niu, Lisa M. Nicholas, Shani Austin-Williams, Emily J. Camm, Megan R. Sutherland, Thomas J. Ashmore, Katie. L. Skeffington, Angela Logan, Susan E. Ozanne, Michael P. Murphy, Dino A. Giussani

**Affiliations:** 1 Department of Physiology, Development and Neuroscience, University of Cambridge, Cambridge, UK; 2 Cambridge Cardiovascular Strategic Research Initiative, Cambridge, UK; 3 Institute of Metabolic Science-Metabolic Research Laboratories, University of Cambridge, Addenbrooke’s Hospital, Cambridge, UK; 4 Medical Research Council Mitochondrial Biology Unit, University of Cambridge, Cambridge, UK; 5 Strategic Research Initiative in Reproduction, Cambridge, UK; 6 Department of Medicine, University of Cambridge, Cambridge, UK

**Keywords:** cardiovascular, fetus, hypoxia, IUGR, mitochondria, oxidative stress, programming

## Abstract

Mitochondria-derived oxidative stress during fetal development increases cardiovascular risk in adult offspring of pregnancies complicated by chronic fetal hypoxia. We investigated the efficacy of the mitochondria-targeted antioxidant MitoQ in preventing cardiovascular dysfunction in adult rat offspring exposed to gestational hypoxia, integrating functional experiments in vivo, with those at the isolated organ and molecular levels. Rats were randomized to normoxic or hypoxic (13%-14% O_2_) pregnancy ± MitoQ (500 μM day^-1^) in the maternal drinking water. At 4 months of age, one cohort of male offspring was chronically instrumented with vascular catheters and flow probes to test in vivo cardiovascular function. In a second cohort, the heart was isolated and mounted onto a Langendorff preparation. To establish mechanisms linking gestational hypoxia with cardiovascular dysfunction and protection by MitoQ, we quantified the expression of antioxidant system, β-adrenergic signaling, and calcium handling genes in the fetus and adult, in frozen tissues from a third cohort. Maternal MitoQ in hypoxic pregnancy protected offspring against increased α_1_-adrenergic reactivity of the cardiovascular system, enhanced reactive hyperemia in peripheral vascular beds, and sympathetic dominance, hypercontractility and diastolic dysfunction in the heart. Inhibition of *Nfe2l2*-mediated oxidative stress in the fetal heart and preservation of calcium regulatory responses in the hearts of fetal and adult offspring link molecular mechanisms to the protective actions of MitoQ treatment of hypoxic pregnancy. Therefore, these data show the efficacy of MitoQ in buffering mitochondrial stress through NADPH-induced oxidative damage and the prevention of programmed cardiovascular disease in adult offspring of hypoxic pregnancy.

## 1 Introduction

Claiming 17.5 million lives annually worldwide, cardiovascular disease (CVD) is a burgeoning epidemic.^1^ Driven by an aging and obese population, CVD prevalence reached the 2030 projection^2^ in 2015. Productivity loss, medical and informal caregiving costs are now expected to exceed $1.2 trillion by 2035.^3^ To significantly reduce total costs associated with CVD, prevention rather than treatment provides the most impactful opportunity. This is particularly relevant to the early origins of CVD, where adverse conditions during pregnancy can increase the cardiovascular risk of adult offspring through a process known as developmental programming.^4^ The window of opportunity for intervention is greatest in the embryonic and fetal periods; therefore, preventative therapeutic administration during the period of pregnancy complications is most appropriate to reduce the programmed risk of CVD in progeny.

Chronic fetal hypoxemia or a sustained reduction in fetal oxygenation is the most common outcome of adverse pregnancy in humans, such as during placental insufficiency, placental infection, preeclampsia, gestational diabetes or maternal obesity.^5,6^ Using preclinical paradigms of disease, several groups have reported that hypoxic pregnancy programs CVD in the adult offspring by increasing oxidative stress in the placenta and in the developing fetal cardiovascular system,^7^.^8–13^ Common adverse consequences of hypoxic pregnancy in adult offspring include enhanced myocardial contractility, impaired ventricular relaxation and cardiac sympathetic dominance.^8,9,14^ Previous studies also report that maternal antioxidant treatment during hypoxic pregnancy, such as with vitamin C or allopurinol, can protect the progeny against programmed CVD.^8,9,15^ However, maternal vitamin C treatment in hypoxic pregnancy is effective only at very high doses incompatible with human translation,^9^ and maternal treatment with allopurinol combats only one of many pro-oxidant pathways.^15^ Since mitochondria are a major site of reactive oxygen species (ROS) production,^16,17^ mitochondria-targeted antioxidant therapy may provide a superior therapeutic strategy.

The mitochondria-targeted antioxidant MitoQ is a bioactive ubiquinone (Coenzyme Q_10_) conjugated to a lipophilic triphenylphosphonium cation, which promotes bioaccumulation 100-500-fold within the mitochondria. Therefore, it is highly effective in combating pathologies resulting from oxidative stress in a range of studies in rodents, and in human clinical trials.^18–21^ We have reported extensively on the benefits and limitations of nontargeted antioxidant therapies during pregnancy ^5,8,9,13,22–25^; however, the use of MitoQ in pregnancy is only just being explored.^26^ Three studies have reported the effects of nanoparticle bound MitoQ (nMitoQ) treatment during hypoxic pregnancy on offspring physiology.^27–29^ Nanoparticle conjugation delivers MitoQ therapy directly to the placenta, while preventing placental barrier crossing and fetal exposure.^30^ Our recent study in sheep administering maternal MitoQ therapy during hypoxic pregnancy showed significant uptake of MitoQ into the fetal tissues and treatment efficacy on the developing fetal cardiovascular system.^7^ Using this ovine model of fetal growth restriction induced by late-onset hypoxic pregnancy, we reported that maternal treatment with MitoQ rescued fetal growth restriction and prevented programmed systemic hypertension in adult female offspring.^7^

Here, we have determined in a rodent model the effects of maternal MitoQ treatment on cardiovascular function in adult offspring of early-onset gestational hypoxia, independent of fetal growth restriction. Integrating in vivo experiments with those at the isolated organ and molecular levels, we tested the hypothesis that maternal MitoQ treatment conveys significant protection against cardiovascular risk in adult rat offspring of hypoxic pregnancy. We explored molecular mechanisms including genes that regulate antioxidant defenses, as well as those involved in cardiac β-adrenergic signaling and calcium-mediated regulation of ventricular contraction and relaxation.

## Materials and Methods

### 2.2 Ethical approval

All animal procedures were performed at a University of Cambridge University Biomedical Services facility. This research was conducted in accordance with the Animals (Scientific Procedures) Act 1986 Amendment Regulations 2012, following ethical review by the University of Cambridge Animal Welfare and Ethical Review Board (AWERB). The experimental design was conducted in accordance with ARRIVE guidelines.^31^

### 2.3 Generation of experimental groups

**Figure 1:**
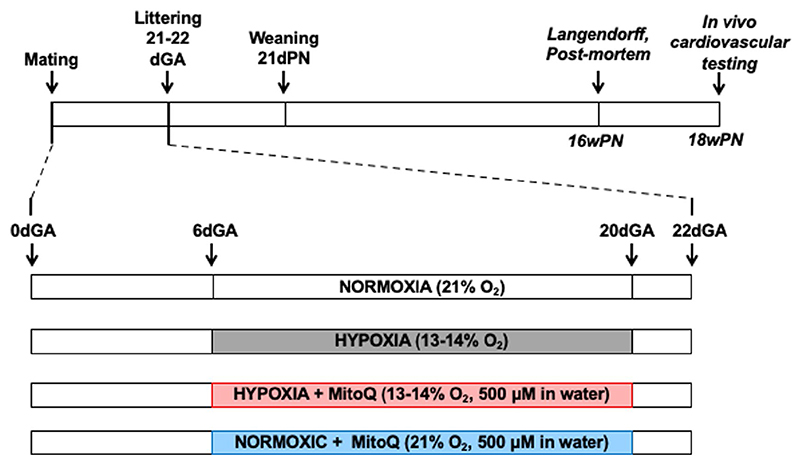
Postnatal experimental design. Pregnant Wistar dams were exposed to normoxia (N; 21% O_2_) or (hypoxia (H; 13%-14% O_2_) from 6 to 20 days gestation (dGA) ± MitoQ (NM and HM, respectively) provided at 500 μM day^−1^ in the maternal drinking water. Offspring were born and maintained in normoxia until 4 months of age. One male per litter was randomly selected for either conscious in vivo cardiovascular testing in chronically instrumented preparations or ex vivo analysis of cardiac function using an isolated heart Langendorff preparation. For in vivo testing, offspring were instrumented with femoral vascular catheters and a femoral transonic flow probe placed in the contralateral leg. Basal and stimulated in vivo cardiovascular function was determined following 4-5 days of postsurgical recovery

Rats were generated as previously described.^9,26^ Briefly, Wistar rats (Charles River, Ltd. Margate, UK) were acclimated for 7-14 days to standard laboratory conditions (60% humidity, 21°C, 12-hour light/dark cycle) with free access to water and food (RM3, Special Diet Services, UK). Nulliparous females (n = 82, 10-12 weeks of age) were individually paired with fertile males (n = 18) and the presence of a copulatory plug was considered pregnancy day 0 (term is 22 days). Pregnant Wistar dams were randomly exposed to normoxia (N; 21% O_2_) or (hypoxia (H; 13%-14% O_2_) with or without 500 μM MitoQ treatment (NM and HM, respectively) provided in maternal drinking water, which was prepared fresh every second day in light-protected bottles. Pregnant rats randomly assigned to hypoxia were placed inside an airtight transparent polyvinyl chloride (PVC) isolator, with air and nitrogen autoregulated to attain 13%-14% O_2_. The isolator housed 12 individual rat cages at any one time in a tranquil environment.^9,26^ Hypoxic pregnancies were exposed to 13%-14% O_2_ from 6 to 20 days gestation (dGA), and pregnant dams randomly allocated to receive MitoQ therapy were treated from 6 to 20 dGA. Exposure to 13%-14% O_2_ during pregnancy leads to a reduction in fetal PaO_2_ levels^32^ similar to that found in complicated human pregnancy,^33^ and human pregnancy at high altitude.^34^ This model of early-onset gestational hypoxia did not affect maternal food intake and has previously been shown not to affect maternal food intake or lead to fetal growth restriction.^8,9,26,35^ Therefore, the effects of hypoxia on programmed cardiovascular dysfunction in adult offspring are independent of changes in maternal nutrition or intrauterine growth restriction. Exposing pregnant rats to hypoxia earlier than day 6 of gestation markedly enhances pregnancy loss.^8^ In all pregnancies, maternal weight gain (g day^-1^), food (g kg d^-1^), water (mL kg d^-1^), and MitoQ intake (mg kg d^-1^) were calculated from 7 to 20 dGA. One cohort of gravid dams (N, n = 14; H, n = 13; HM, n = 8, and NM, n = 12) were randomly allocated to undergo CO_2_ euthanasia and cervical dislocation at 20 dGA. The placenta, maternal liver, fetal liver, and the fetal heart were snap-frozen in liquid nitrogen and stored at −80°C until molecular analysis. A second cohort of gravid dams (N, n = 10; H, n = 8; HM, n = 8, and NM, n = 9) was allowed to spontaneously litter, and on postnatal day (dPN) 2, litter size was decreased to eight pups to standardize maternal care. Pups were weaned at 21 dPN, then, sex-split at 35 dPN. At 16 weeks (wPN), one male adult from each litter was randomly allocated to either ex vivo cardiac function testing via an isolated Langendorff preparation (N, n = 6; H, n = 8; HM, n = 6, and NM, n = 8) or euthanasia and tissue collection. Hearts were isolated from one male adult per litter, snap-frozen in liquid nitrogen and stored at −80°C until molecular analysis (N, n = 8; H, n = 6; HM, n = 7, and NM, n = 9). At 18wPN, another male adult per litter was randomly allocated to surgical-instrumentation and in vivo cardiovascular testing (Figure 1; N, n = 10; H, n = 8; HM, n = 8, and NM, n = 9). To control for sex and within-litter variation, placental, fetal, and adult outcomes were quantified in one male offspring per litter. All other tissues and tissues from female offspring were allocated to other studies.

### 2.4 MitoQ tissue uptake

MitoQ was measured in the placenta, maternal and fetal liver, and fetal heart at 20 dGA, as previously reported.^36^ Frozen tissue (50 mg) was homogenized in 300 μL of 50 mM Tris buffer (pH 7.0) with a Bullet blender homogenizer (Next Advance, New York, USA). A 600 μL 95% acetonitrile (Romil, Cambridge, UK) and 0.1% formic acid (FA; Sigma-Aldrich, Gillingham, UK) was added to the homogenate. The samples were spiked with an internal standard (IS; 0.5 μM *d_3_*-MitoQ in ethanol (ethanol Sigma-Aldrich, Gillingham, UK), vortexed, and centrifuged at 16 000 *g* for 10 minutes. The supernatant was transferred to a fresh Eppendorf tube, 500 μL more of 95% ACN/0.1% FA was added to the pellet, and the homogenization and centrifugation steps repeated. The two supernatants were then combined, centrifuged again (10 minutes at 16 000 *g*), the supernatant transferred to a fresh Eppendorf tube and left to dry overnight in a Savant SpeedVac. The following day 170 μL of 20% ACN/0.1% FA solution was added to the pellet and the solution mixed with a vortex, then, centrifuged (10 minutes, 16 000 *g*) before being filtered (with a 1 mL disposable syringe and 0.22 μm PVDF filters; Millipore, Watford, UK) into a mass spectrometry autosampler vial (1.5 mL salinized, Thermo Fisher Scientific, Loughbrough, UK). Control tissues were spiked with known amounts of MitoQ [in pmol: 0 (not spiked with IS but given an equivalent volume of ethanol), 1, 19, 25, 100, and 500] and the protocol above was repeated to generate a standard curve. MitoQ concentration was analyzed by liquid chromatography-tandem mass spectrometry (LC/MS/ MS) using an I-class Acquity UPLC attached to a Xevo TQ-S triple quadruple mass spec (both Waters, Milford, USA), and expressed relative to the IS by multiple reaction monitoring using the transition 583 > 441. The results were analyzed with MassLynx software.

### 2.5 Measurement of postnatal growth

Pups were weighed daily between 2 and 7 dPN, every-other-day until weaning (21 dPN), and weekly from weaning to 16 wPN. Fractional growth rate (FGR, g day^-1^) was calculated pre- and postweaning (2-21 dPN and 3-16 wPN, respectively).

### 2.6 Surgical instrumentation for in vivo cardiovascular testing

Adult male rats were acclimated to single housing and handling 1 week prior to surgery. Inhalation anesthesia was induced with 2.5%-5.0% isoflurane (Henry Schein, UK) in 2 L min^−1^ 100% O_2_ and maintained with 2.0%-2.5% isoflurane via nosecone throughout the surgical procedure. The rat was placed on a homeothermic platform, a rectal probe inserted (Harvard Apparatus, UK), and eyes lubricated with ointment (Puralube, Dechra, UK). Subcutaneous 5.0 mg kg^−1^ carprofen and 10 mg kg^−1^ enrofloxacin (both Henry Schein, UK) were given subcutaneously, and bilateral inguinal regions shaved, washed with dilute chlorhexidine (Hibiscrub, Henry Schein, UK), and swabbed with 70% ethanol (Sigma-Aldrich, UK). Rats were wrapped in sterile Press’n’ Seal (Baco, UK) and transferred to a sterile draped SurgiSuite Surgical Platform (Kent Scientific, UK). With the rat placed supine, a 1.0 cm incision was made at the inguinal crease, bilaterally, and a pocket blunt dissected craniodorsally toward the scapulae. The rat was placed prone, a 1.0 cm mid-scapular incision made and pocket blunt dissected, and Rochester forceps passed from the scapulae to the inguinal incisions for catheter and flow probe cable exteriorization. Vascular access harness ports (Instech Laboratories, Inc, US) were connected to custom-made 1 to 3 Fr funnel tip polyurethane catheters (SAI Infusion, Ltd., US) and prefilled with 100 U mL^−1^ heparinized sterile saline. The left femoral artery and vein were ligated with a 4.0 silk suture tied below the catheterization site. Catheters were introduced into the femoral artery and vein and advanced into the abdominal aorta and inferior vena cava, respectively. Catheters were secured with a 4.0 silk suture proximal to the insertion site, and immediately distal to the 1 Fr joint. Catheters were looped and secured to the *gracilis* muscle, and excess catheter looped at the scapular access point. A custom-made perivascular flow probe (0.7PSL Back Exit NanoProbe, Transonic, US) was implanted around the femoral artery of the contralateral leg. The flow probe cable was secured to the *gracilis* with a 4.0 silk suture, and excess cable tucked at the scapular access point. The flow probe connector was secured with a rigid cuff, and surgical wounds closed with 6.0 Prolene interrupted sutures (Ethicon, UK). Anesthesia was reduced to 1.5% isoflurane, while femoral blood flow was monitored with a TS420 flow box (Transonic, US), and arterial blood pressure via fluid-filled pressure transducer through a 4-channel bridge amp and 8-channel PowerLab in LabChart 8 (all, ADInstruments, US). A dual-channel vascular access harness was then secured, and rats provided with homeothermic support during recovery. Vascular catheters were flushed daily to maintain patency, and carprofen provided for 2 days postsurgery. Rats were acclimated to the testing cage for 30 minutes daily, for 4-5 days.

### 2.7 Conscious In vivo cardiovascular testing

Four to five days postsurgery, access ports were swabbed with 70% ethanol, a two-channel vascular access tether (Instech, US) was prefilled with 100 U mL^−1^ heparinized sterile saline and connected to the vascular access harness to allow simultaneous drug infusion and cardiovascular recording. Following 30-minute acclimation to the testing cage, basal heart rate, arterial blood pressure, and femoral blood flow were recorded for 30 minutes. Changes in arterial blood pressure and femoral blood flow were recorded in response to intravenous infusion of the α_1_-adrenergic agonist phenylephrine (PE, Sigma-Aldrich, UK) administered in a randomized order (5.0, 10.0, 20.0, 40.0, and 80.0 μg kg^−1^,) in 100 μL 0.9% saline, which has been shown to have no effect on hemodynamics.^37^ Arterial blood pressure and femoral blood flow were allowed to return to a stable 2-minute baseline between doses. Basal and stimulated heart rate, blood pressures and blood flow response post-PE infusions were measured. Peak changes from baseline were calculated from the raw data.^38^ To ascribe to National Centre for the Replacement Refinement & Reduction of Animals in Research guidelines, following cardiovascular testing instrumented rats were used in subsequent studies not described herein and not related to this study.

### 2.8 Ex vivo assessment of cardiac function using the Langendorff preparation

Adult male rats randomly allocated for isolated heart functional studies were euthanized by CO_2_ inhalation and posterior cervical dislocation. The heart was isolated and mounted onto a Langendorff preparation.^15,22^ In brief, a re-circulating Krebs-Henseleit bicarbonate buffer solution containing (mmol L^−1^) 120 NaCl, 4.7 KCl, 1.2 MgSO2.7 H_2_O, 1.2 KH2PO4, 25 NaHCO_3_, 10 glucose, and 1.3 CaCl_2_.2H_2_O was filtered (5 μm cellulose nitrate; Millipore, Bedford, MA, USA) and bubbled with an O_2_:CO_2_ (95:5) gas mixture at 37°C. Isolated hearts were perfused retrogradely through the aorta at a constant 100 cm H_2_O pressure. A small flexible nonelastic balloon made of clingfilm was connected to a rigid catheter filled with distilled water and inserted into the left ventricle through the left atrium. The volume of the balloon was adjusted to obtain a left ventricular (LV) end diastolic pressure (LVEDP) recording between 5 and 10 mmHg.^15,22^ After 15 minutes of stable baseline, heart rate (HR), LV systolic pressure (LVSP), and LVEDP were recorded. Basal LV developed pressure (LVDP) was calculated as the difference between LVSP-LVEDP. The maximum and minimum first derivatives of the LV pressure (dP/dt_max_ and dP/dt_min_) were calculated. Contractility index, an index of systolic function, was calculated as the value for dP/dt_max_ normalized to mean pressure at the point of dP/ dt_max_.^39^ The cardiac responsiveness to the muscarinic agonist Carbachol (carbamylcholine chloride, Sigma-Aldrich Co. Ltd, Poole, UK; range, 10^-10^ to 10^-6^ M) and the β_1_ adrenoreceptor agonist Isoprenaline ((-)-Isoprenaline (+)-bitartrate salt, Sigma-Aldrich Co. Ltd, Poole, UK; range, 10-11 to 10-7 M) were investigated, as described before.^15,22^ The ratio of the maximal LV contractile (ionotropic) response to Isoprenaline relative to that of Carbachol is an established measure of cardiac sympathetic dominance.^8,15,22^

### 2.9 Quantitative PCR

Molecular analyses were conducted in accordance with MIQE guidelines.^40^ Snap-frozen hearts were ground, and total RNA extracted with the RNeasy Mini RNA purification kit with an on-column DNA digest step (Qiagen GmbH, Hilden, Germany) according to the manufacturer’s instructions. mRNA concentration and integrity were determined with a NanoDrop Spectrophotometer (Thermo Fischer Scientific, Waltham, MA, USA). Samples of acceptable purity (260/280 absorbance > 1.9, and 260/230 absorbance > 1.6) were reverse transcribed with RevertAid First Strand complementary DNA (cDNA) synthesis kit (Fermentas, Vilnius, Lithuania) in 20 μL reaction volume with 200 ng total mRNA. cDNA was synthesized on a Verity Thermal Cycler (Applied Biosystems, UK) under the following conditions: 25°C for 10 minutes, 42°C for 60 minutes, 70°C for 5 minutes, and cooled to 4°C and stored at 20°C until quantitative PCR (qPCR) analysis. Transcript abundance was determined by singleplex qPCR amplification in 384-well plates on a QuantStudio Flex 7 Real-Time PCR Instrument (PE Applied Biosystems, Foster City, CA, USA) in duplicate, including interplate, positive and negative controls. Reactions were run with a final volume of 6.0 μL containing 3.0 μL SYBR Green Master Mix (PE Applied Biosystems, Foster City, CA), 0.36 μL each forward and reverse primers (Sigma-Aldrich, UK) at a final concentration of 300 nM, 1.64 μL molecular grade H_2_O, and 2.5 ng cDNA template under the following conditions: 50°C for 2 minutes and 95°C for 10 minutes, 40 cycle-repeats of 95°C for 15 seconds and 60°C for 1 minute. Rat target genes involved in oxidative stress and cardiac calcium regulation were analyzed: superoxide dismutase 2 (*Sod2*), catalase (*Cat*), glutathione peroxidase 1 (*Gpx1*), nuclear factor, erythroid-like 2 (*Nfe2l2*), cyclic adenosine monophosphate-dependent protein kinase (*Camk2d*), phospholamban (*Pln*), sarcoplasmic/endoplasmic reticulum Ca_2_^+^ transporting ATPase 2A (*Atp2a2*), ryanodine receptor 2 (*Ryr2*) (Table S1). The stability of three potential reference genes across experimental groups were determined for each tissue: beta actin (*Actb*), peptidylprolyl isomerase A (*Ppia*), and hypoxanthine phosphoribosyltransferase 1 (Hprt1) (Table S1). GenBank rat sequences were analyzed for target genes with Primer Express Software (Applied Biosystems). Primer locations were determined, and sequences BLAST searched to confirm target-specific oligonucleotide sequences. PCR amplicons were verified as previously described.^41^ Pooled cardiac cDNA (2 μL from each sample) was diluted twofold for standard curve optimization, and to determine amplification efficiencies for all target genes and potential reference genes, were calculated from the slopes of the standard curves (Table S2). Reference gene expression stability from pooled samples were assessed with GeNorm.^42^ qPCR mRNA expression was calculated, as previously described.^43–45^ Briefly, the geometric mean (GEO) of the two most stable reference genes was calculated for each age (fetal: *Ppia* and *Hprt1*; adult: *Actb* and *Hprt1*) across the experimental groups.^46^ The threshold cycle (Ct) for target gene (TG) mRNA expression levels were calculated relative to the reference geometric mean with a mathematical model which accounts for variation in transcript amplification efficiency.^47^ The standard error of the mean (SEM) was calculated for each group and a 95% confidence interval (CI), as previously described.^43–45^ Results are reported as fold-change with 95% CI relative to either Normoxic (H, HM, and NM) or Hypoxic (HM) for fetal and adult tissues.

### 2.10 Statistical analysis

Appropriate power calculations derived from previous data sets were performed for each component of the program of work to determine the minimum sample size required to detect statistically significant differences (*P* < .05). Wherever possible, scientists measuring outcomes were blinded to treatments. Data are presented as the mean ± SEM. Data were analyzed in JMP 12 (SAS Institute Inc, Cary, North Carolina, USA). Distribution was verified with the Shapiro-Wilk test, and nonparametric data were log transformed to approximate a normal distribution where necessary. The effects of hypoxia and MitoQ treatment were analyzed by factorial analysis of variance (two-way ANOVA), student *t* test where appropriate. Tukey’s post hoc testing was conducted where appropriate. To directly assess the difference in mRNA expression of hypoxia- and MitoQ-treated experimental groups to normoxic and untreated, expression was considered statistically different at the 5% level where 95% CIs do not include 1.0.

## 3 Results

### 3.1 MitoQ crosses the placenta in control and hypoxic pregnancy

Hypoxic pregnancy did not affect maternal intake of food or water, but it reduced maternal fractional weight gain and litter size (Table S3). Hypoxic pregnancy did not affect pup weight at postnatal day 2 or weaning (21 dPN), but it reduced adult offspring weight at 16 wPN. Accordingly, the pup postweaning fractional weight gain was reduced by hypoxic pregnancy between 3 and 16 weeks (Table S3). Maternal MitoQ treatment produced a similar daily intake of the drug in normoxic and hypoxic pregnancy (Figure S1 and Table S3). Maternal MitoQ crossed the placenta and was taken up in fetal tissues to similar extents in both normoxic and hypoxic pregnancy (Figure S1). However, maternal MitoQ did not affect the reduction in fractional weight gain in hypoxic dams during pregnancy or in offspring of hypoxic pregnancy postweaning (Table S3). The absolute and relative weights for most organs were comparable across groups in adult offspring. However, the absolute brain weight was reduced in adult offspring of hypoxic pregnancy, irrespective of MitoQ treatment (Table S4).

### 3.2 MitoQ normalizes in vivo cardiovascular dysfunction in adult offspring of hypoxic pregnancy

**Figure 2:**
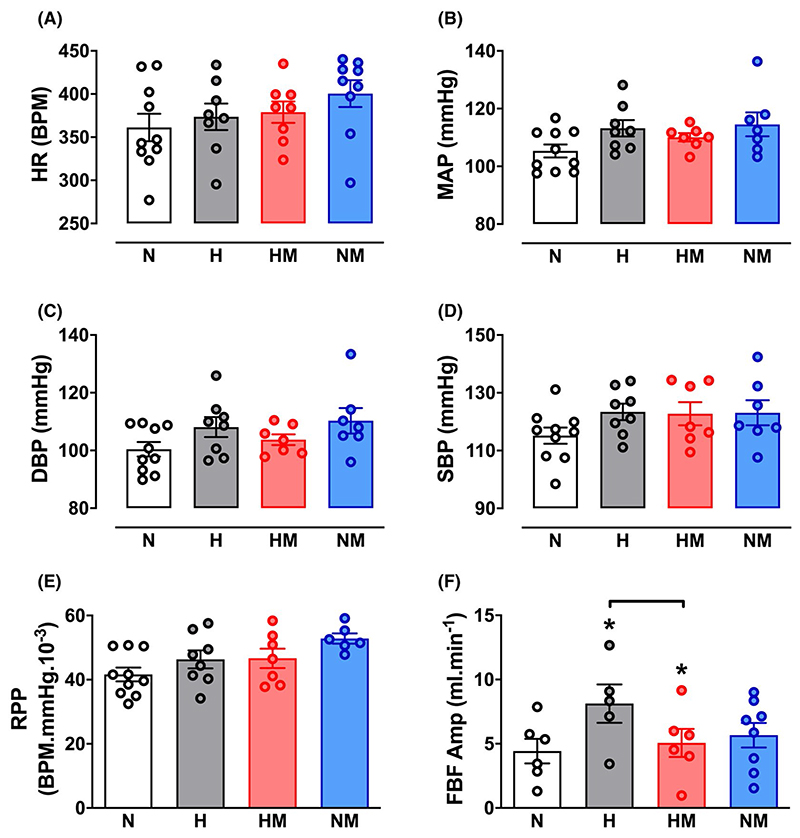
Effect of hypoxic pregnancy and MitoQ on basal cardiovascular function in the adult offspring. Values are means ± SEM for heart rate (HR; A), mean arterial pressure (MAP; B), systolic blood pressure (SBP; C), diastolic blood pressure (DBP; D), the rate-pressure product (RPP; E), and femoral blood flow amplitude (FBF Amp; F) in adult offspring of normoxic (N, n = 6-10), hypoxic (H, n = 5-8), hypoxic treated with MitoQ (HM, n = 6-8), or normoxic treated with MitoQ (NM, n = 6-9) pregnancies. Statistical differences are (*P* < .05): *main effect of hypoxia; hypoxia x MitoQ interaction indicated by brackets (Two-Way ANOVA with Tukey’s post hoc comparison)

**Figure 3:**
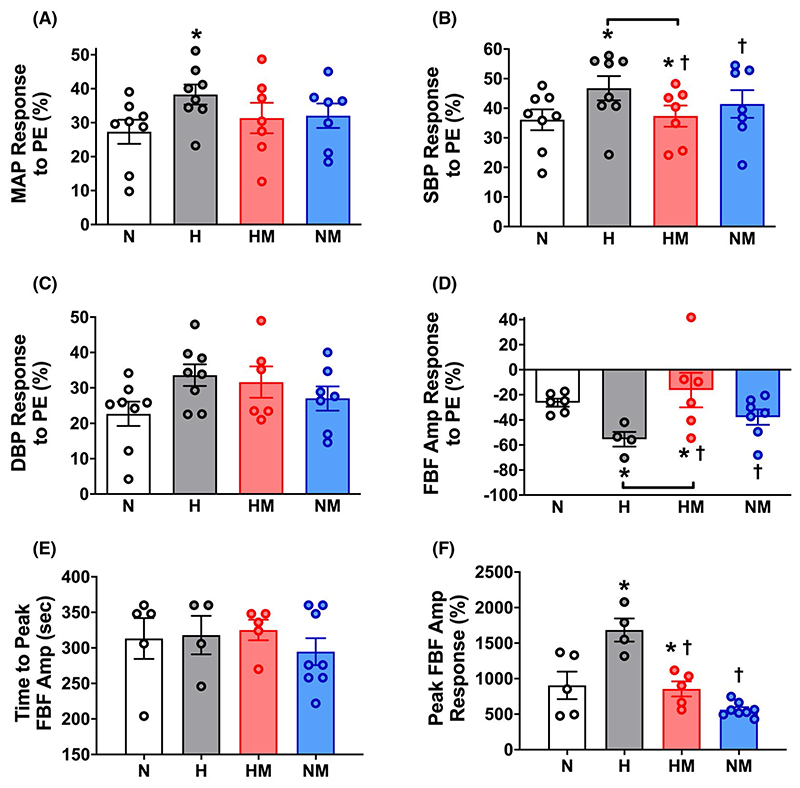
Effect of hypoxic pregnancy and MitoQ on stimulated cardiovascular function in the adult offspring. Values are means ± SEM. for the percent change from baseline in mean arterial pressure (MAP; A), systolic blood pressure (SBP; B), diastolic blood pressure (DBP; C), and the fall in the amplitude of femoral blood flow (FBF Amp; D) in response to in vivo treatment with an intravenous dose of 80 μg kg^-1^ phenylephrine (PE) in conscious adult offspring of normoxic (N, n = 6-8), hypoxic (H, n = 4-8), hypoxic treated with MitoQ (HM, n = 6-7), or normoxic treated with MitoQ (NM, n = 7) pregnancies. Panels E and F show the reactive hyperemic response in femoral blood flow post-PE infusion in terms of time to peak and the peak amplitude of the femoral dilator response, respectively. Statistical differences are (*P* < .05): * main effect of hypoxia; † main effect of MitoQ; hypoxia x MitoQ interaction indicated by brackets (Two-Way ANOVA with Tukey’s post hoc comparison)

Hypoxic pregnancy did not affect basal arterial blood pressure or heart rate, but it increased basal femoral blood flow and the magnitude of the blood pressure and femoral vasoconstrictor responses to exogenous treatment with phenylephrine in the adult offspring when measured in vivo (Figures 2 and 3). Following phenylephrine-induced femoral vasoconstriction, there was a reactive hyperemic response in the femoral vascular bed, which was significantly greater in adult offspring of hypoxic compared with normoxic pregnancy (Figure 3). Maternal MitoQ treatment in hypoxic pregnancy normalized the programmed changes in basal and stimulated cardiovascular function in adult offspring of hypoxic pregnancy. Maternal MitoQ treatment in normoxic pregnancy did not alter in vivo cardiovascular function of adult offspring (Figures 2 and 3).

### 3.3 MitoQ protects against cardiac dysfunction in adult offspring of hypoxic pregnancy

**Figure 4:**
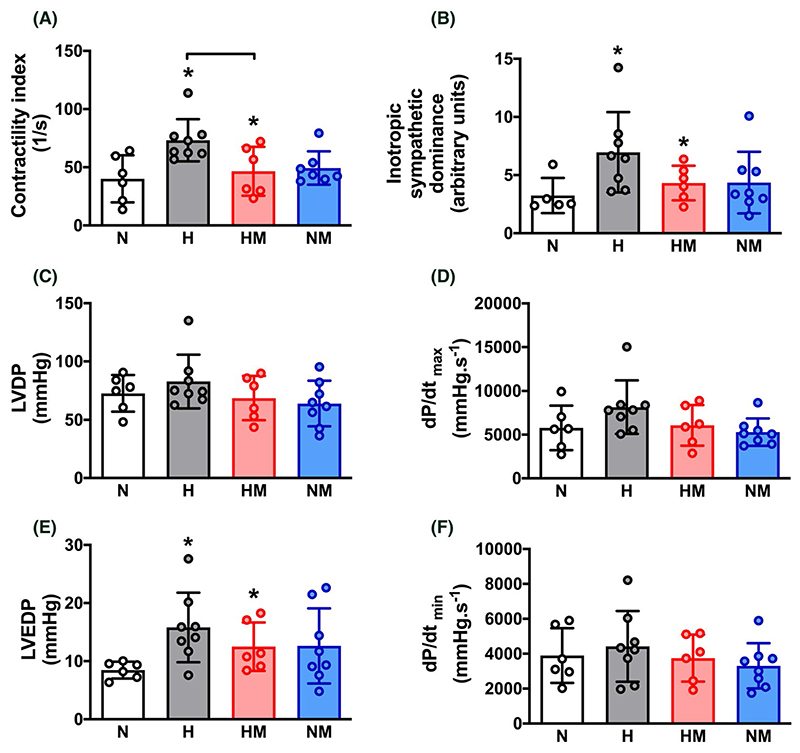
Effect of hypoxic pregnancy and MitoQ on isolated cardiac function in the adult offspring. Values are means ± SEM. for the contractility index (A), the inotropic sympathetic dominance (B), left ventricular developed pressure (LVDP; C), the maximum first derivative of the left ventricular pressure (dP/dtmax; D), left ventricular end diastolic pressure (LVEDP; E), and the minimum first derivative of the left ventricular pressure (dP/dtmin; F) in adult offspring of normoxic (N, n = 5-6), hypoxic (H, n = 8), hypoxic treated with MitoQ (HM, n = 6), or normoxic treated with MitoQ (NM, n = 7-8) pregnancies. The inotropic sympathetic dominance was calculated as the ratio of the LVDP response to a maximal dose of isoprenaline relative to a maximal dose of carbachol. Statistical differences are (*P* < .05): * main effect of hypoxia; hypoxia x MitoQ interaction indicated by brackets (Two-Way ANOVA with Tukey’s post hoc comparison)

Hearts isolated from adult offspring of hypoxic pregnancy showed increased contractility index, inotropic sympathetic dominance and an elevated left ventricular end diastolic pressure (Figure 4). These effects of hypoxic pregnancy did not occur in hearts isolated from adult offspring of hypoxic pregnancy treated with MitoQ (Figure 4). Maternal MitoQ treatment in normoxic pregnancy did not affect ex vivo cardiac function (Figure 4).

### 3.4 MitoQ protects against fetal cardiac mitochondrial oxidative stress and normalizes antioxidant enzyme gene expression in hearts of adult offspring of hypoxic pregnancy

**Figure 5:**
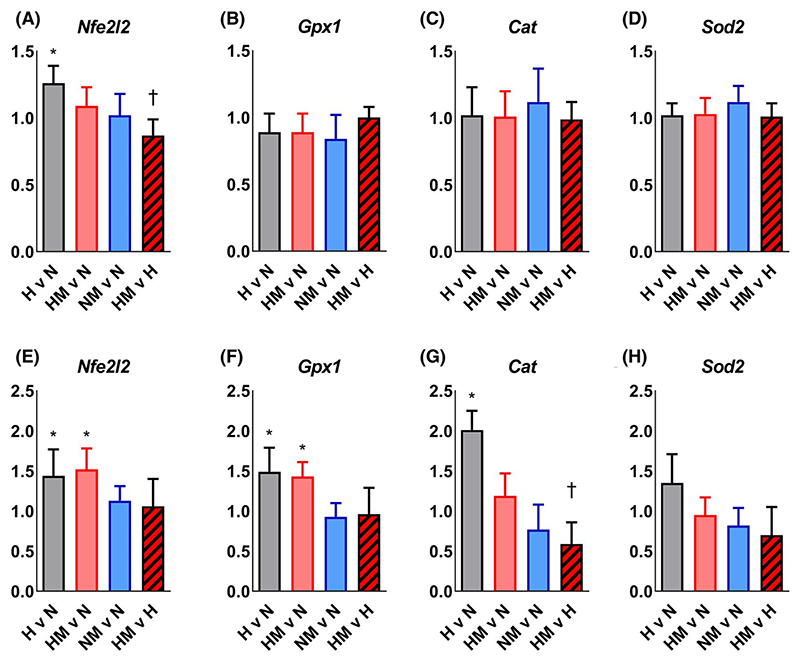
Effect of hypoxic pregnancy and MitoQ on mitochondrial oxidative stress and endogenous antioxidant mRNA expression in the heart of fetal and adult offspring. Values are for the mRNA expression in hearts of fetal (A-D) and adult (E-H) offspring of hypoxic (H; grey, n = 5-6), hypoxic MitoQ (HM; red, n = 6-7), and normoxic MitoQ (NM; blue, n = 5-9) relative to normoxic (N, n = 6-8) groups, and of HM relative to H (striped) groups. Data are normalized to the geometric mean of the two most stable reference genes and are reported as fold change (95% Confidence Interval, CI). Where CIs do not include 1.0, mRNA expression is statistically different from N (*: H, HM, and NM), or H (†: HM) at the 5% level

Hypoxic pregnancy increased *Nfe2l2* expression without affecting antioxidant enzyme mRNA expression in fetal hearts (Figure 5A–D). In adulthood, the hypoxia-induced increase in cardiac *Nfe2l2* expression persisted, and hypoxic pregnancy also increased cardiac *Gpx1* and *Cat* expression (Figure 5E–G). Maternal MitoQ treatment of hypoxic pregnancy normalized the expression of *Nfe2l2* in the fetal heart (Figure 5A) and *Cat* expression in the adult heart (Figure 5G). However, the increased *Nfe2l2* and *Gpx1* expression in hearts of adult offspring of hypoxic pregnancy was not influenced by MitoQ (Figure 5E and F).

### 3.5 MitoQ preserves adaptive molecular responses in calcium regulation without affecting β-adrenergic signaling in hearts of fetal and adult offspring of hypoxic pregnancy

**Figure 6:**
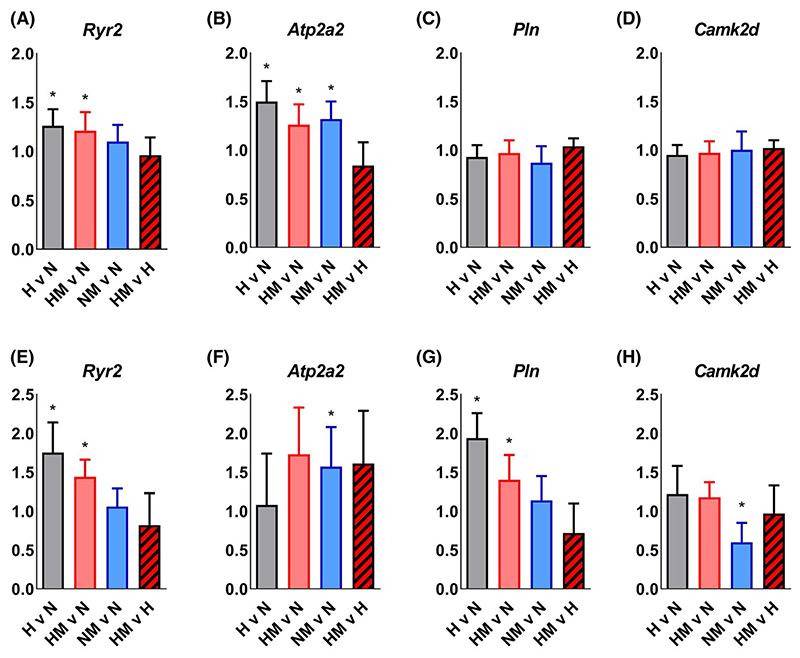
Effect of hypoxic pregnancy and MitoQ on calcium regulation and β-adrenergic signaling mRNA expression in the heart of fetal and adult offspring. Values are for the mRNA expression in hearts of fetal (A-D) and adult (E-H) offspring of hypoxic (H; grey, n = 5-6), hypoxic MitoQ (HM; red, n = 6-7), and normoxic MitoQ (NM; blue, n = 5-9) relative to normoxic (N, n = 6-8) groups, and of HM relative to H (striped) groups. Data are normalized to the geometric mean of the two most stable reference genes and are reported as fold change (95% Confidence Interval, CI). Where CIs do not include 1.0, mRNA expression is statistically different from N (*: H, HM, and NM) at the 5% level

Hypoxic pregnancy increased *Ryr2* and *Atp2a2* expression, without affecting the expression of *Pln* and *Camk2d* in fetal hearts (Figure 6A–D). In adulthood, the hypoxia-induced increase in cardiac *Ryr2* expression, but not *Atp2a2*, persisted and hypoxic pregnancy also induced an increase in cardiac *Pln* expression (Figure 6E–G). The increased *Ryr2* and *Atp2a2* expression in hearts of fetal offspring of hypoxic pregnancy were not affected by MitoQ (Figure 6A and B). The increased *Ryr2* and *Pln* expression in hearts of adult offspring of hypoxic pregnancy were not affected by MitoQ (Figure 6E and G). Hearts of fetal offspring of normoxic pregnancy treated with MitoQ showed increased *Atp2a2* expression relative to hearts of fetal offspring of untreated normoxic pregnancy. Hearts of adult offspring of normoxic pregnancy treated with MitoQ showed increased *Atp2a2* expression and reduced *Camk2d* expression relative to hearts of adult offspring of untreated normoxic pregnancy.

## 4 Discussion

These data show that maternal MitoQ treatment in a rodent model of early-onset gestational hypoxia independent of fetal growth restriction protects adult offspring against increased α_1_-adrenergic reactivity of the cardiovascular system, enhanced reactive hyperemia in peripheral vascular beds, and sympathetic dominance, myocardial hypercontractility and diastolic dysfunction in the heart. Increased gene expression of erythroid 2-related factor 2 (*Nfe2l2*) in the fetal heart links the oxidative stress response to gestational hypoxia. Greater *GPx1* and *Cat* expression in the hearts of adult offspring of hypoxic pregnancy supports cardiac antioxidant compensatory responses. Increased cardiac *Ryr2* and *Atp2a2* expression shows that gestational hypoxia induces adaptive changes in calcium regulation in the fetal heart, some of which are maintained to adulthood. Inhibition of the *Nfe2l2*-mediated oxidative stress response in the fetal heart, with preservation of calcium regulatory responses in the hearts of fetal and adult offspring, links molecular mechanisms to the protective actions of MitoQ in early-onset hypoxic pregnancy.

Although maternal treatment with conventional antioxidants in hypoxic pregnancy has proven protective on offspring cardiovascular function,^5,8,9,13,22–25^ their beneficial effects are limited because they cannot readily penetrate the mitochondria, a primary source of ROS production.^16,17^ Conventional antioxidants do not accumulate in the mitochondria, so they may affect ROS levels and redox signals throughout the cell, possibly disrupting cell signaling pathways.^48–52^ MitoQ moves through lipid bilayers, independent of an active uptake mechanism, due to the lipophilic triphenylphosphonium cation. The molecular characteristics of MitoQ allow it to circumvent previously reported barriers to antioxidant efficacy as, driven by the plasma and mitochondrial membrane potentials, it targets the mitochondria accumulating several 100-fold within the organelle.^16^ In the mitochondria, the superoxide anion generated by the respiratory chain is used both for physiological function and converted to hydrogen peroxide, which in conjunction with ferrous iron initiates damaging lipid peroxidation at the unsaturated fatty acid-rich inner mitochondrial membrane. Within the mitochondria, the ubiquinone moiety of MitoQ is reduced to the antioxidant ubiquinol moiety and recycled by succinate dehydrogenase, providing a self-sustaining antioxidant pool that inhibits lipid peroxidation downstream of superoxide and hydrogen peroxide production.^7,16,17^ Therefore, MitoQ limits oxidative stress while maintaining many aspects of ROS signaling pathways, which contribute to the regulation of physiological function. Data in the present study show that oral, maternal MitoQ treatment crosses the rat placenta and accumulates in fetal tissues, in both control and hypoxic pregnancy, at therapeutic levels.^16,17^ Within the fetal heart, MitoQ normalizes the hypoxia-mediated increase in Nfe2l2/ Nrf2 expression, confirming its efficacy in protecting against mitochondria-mediated oxidative stress in offspring of complicated pregnancy.^53^

Evidence derived from studies of adult men at high altitude has established that exposure to chronic hypoxia induces endothelial dysfunction mediated in part through heightened adrenergic vasoconstrictor signaling.^54^ This effect of chronic hypoxia on adrenergic pathways innervating peripheral blood vessel resistance can be triggered early in life, leading to heightened adrenergic vasoconstrictor signaling in the embryonic or fetal periods,^55–58^ as well as permanently in adult offspring.^13,59,60^ For instance, chronically hypoxic fetal sheep have enhanced plasma catecholamine and α_1_-adrenergic peripheral vasoconstrictor responses to acute stress.^55^ Similarly, adult rat and sheep offspring of hypoxic pregnancy show evidence of endothelial dysfunction and sympathetic dominance despite being reared under normoxic conditions.^13,61^ It is also accepted that there is an interaction between these effects of chronic hypoxia on heightened adrenergic vasoconstrictor signaling and endothelial dysfunction with oxidative stress. For example, chronic hypoxia-induced oxidative stress decreases nitric oxide (NO) bioavailability, thereby enhancing sympathetic vasoconstrictor influences on peripheral resistance circulations.^13^ The present study shows that early-onset gestational hypoxia programs greater pressor and femoral vasopressor responses to phenylephrine in adult offspring, consistent with the effects of chronic hypoxic programming of heightened adrenergic vasoconstrictor signaling. Further, data in the present study show that maternal MitoQ treatment in hypoxic pregnancy normalized this effect, suggesting that the mechanistic interaction between chronic hypoxia in utero and heightened adrenergic vasoconstrictor signaling programmed in adult offspring involves mitochondria-derived oxidative stress. Evidence also supports that chronic hypoxia during pregnancy can program an increase in NO bioavailability in adult offspring, offsetting the effects of chronic hypoxia-enhanced vasoconstrictor pathways and impaired endothelial NO-dependent vasodilation.^7,13,62^ Data in the present study are also consistent with this thesis, since adult offspring of hypoxic pregnancy had greater basal blood flow and an enhanced reactive hyperemic response in the femoral vascular bed, effects which have been ascribed, in part, to enhance NO signaling.^62–65^ Consequently, maternal MitoQ treatment in hypoxic pregnancy, by ameliorating mitochondria-derived oxidative stress in the fetus and preventing the programing of sympathetic vasoconstrictor hyperreactivity in adult offspring, also prevented the need for programmed compensatory increases in both antioxidant defenses and NO bioavailability in the cardiovascular system.

Previous studies have also confirmed that embryonic and fetal development under chronic hypoxic conditions can program increased cardiovascular responsiveness to sympathetic stimulation and myocardial contractility in adult offspring, enhancing cardiac systolic function.^8,15,66–68^ The term cardiac sympathetic dominance describes an increased ratio of the maximal cardiac response to isoprenaline relative to that of carbachol.^8,15,22,39,67,68^ It has been suggested that this cardiac sympathetic dominant response is necessary to overcome programmed increases in peripheral vasoconstriction and cardiac afterload, thereby maintaining cardiac output in adult offspring of hypoxic pregnancy.^8,15,39,67,68^ However, data in the present study reveal that a programmed increase in cardiac sympathetic dominance can occur in the absence of systemic hypertension in adult offspring of hypoxic pregnancy. This highlights that a cardiac sympathetic dominant phenotype can be programmed directly by adverse intrauterine conditions, and not as secondary changes in the peripheral vasculature or blood pressure of adult offspring, as is the case for maternal obesogenic pregnancy.^69^ Additionally, data in the present study link the programmed increase in cardiac contractility with changes in the molecular regulation of calcium physiology in hearts of fetal and adult offspring of hypoxic pregnancy. The ryanodine receptor 2 (RYR2) is a protein found primarily in cardiac muscle, encoded by the *Ryr2* gene.^70^ The *Atp2a2* gene encodes a SERCA Ca^2+^- ATPase, an intracellular pump located in the sarcoplasmic or endoplasmic reticula of cardiac muscle cells.^71^ The *Pln* gene encodes phospholamban, an integral membrane protein regulating the Ca^2+^ pump in cardiac muscle cells.^72^ The *Camk2d* gene encodes the enzyme calcium/calmodulin-dependent protein kinase type II delta chain, which is implicated in calcium and β-adrenergic receptor signaling in the heart.^73^ We show that early-onset gestational hypoxia increased *Ryr2* and *Atp2a2* expression, but not *Pln* or *Camk2d* in fetal hearts. By adulthood, cardiac *Ryr2* and *Pln* expression, but not *Atp2a2* and *Camk2d*, were increased in hearts of offspring of hypoxic pregnancy. Further, data in the present study show an interaction between mitochondria-derived oxidative stress in fetal life and cardiac systolic and diastolic dysfunction in adult offspring, as maternal MitoQ treatment of hypoxic pregnancy normalized the cardiac phenotype. Conversely, there appears to be no interaction between mitochondria-derived oxidative stress in fetal life and alterations in the molecular regulation of cardiac calcium in the hearts of fetal and adult offspring of hypoxic pregnancy. Therefore, the data implicate mitochondria-derived oxidative stress mechanisms that program cardiac dysfunction in adult offspring via pathways independent of the molecular regulation of calcium.

### 4.1 Perspective

The concept of the developmental origins of health and disease stems from human population studies, which show an association between small body size at birth and increased risk of cardiovascular disease in adulthood.^74^ These findings triggered “the fetal origins hypothesis,” which proposed that cardiovascular disease in later life originates from adaptations that the fetus makes when development is compromised by adverse intrauterine conditions. These compensatory responses, which can include the slowing of fetal growth, permanently change the structure and function of organs and systems in the fetus to maximize survival and the successful outcome of pregnancy, but claiming trade-offs that increase susceptibility to dysfunction in adulthood.^75^ Since, there has been discussion about whether fetal growth restriction is a necessary prerequisite for programming cardiovascular risk, or a common symptom that can be used as a surrogate measure for a suboptimal in utero environment.^76^ In contrast to many studies in small and large animal models that report that late-onset hypoxic pregnancy leads to fetal growth restriction and cardiovascular dysfunction in the offspring,^5,7–13^ the present study reports two findings that dissociate both outcomes. These data show that early-onset gestational hypoxia programs cardiovascular dysfunction in adult offspring independent of neonatal weight. Further, maternal MitoQ treatment in hypoxic pregnancy protected against programmed cardiovascular dysfunction in adult offspring without a beneficial effect on newborn weight. Therefore, these data strongly support that low birth weight is not indispensable for cardiovascular programming. Rather, the data highlight that developmental hypoxia per se can increase the risk of cardiovascular disease in the offspring, but whether fetal growth restriction occurs depends on the timing, severity, and duration of the challenge.^77^

## Supplementary Material

Supporting information Additional Supporting Information may be found online in the Supporting Information section.

## Data Availability

The data that support the findings of this study are available herein. Additional relevant data can be requested from the corresponding author.

## Abbreviations

ARRIVE: guidelines, animal research: reporting of in vivo experiments
AWERB: Animal Welfare and Ethical Review Board of the University of Cambridge
CaCl_2_: calcium chloride
dGA: days gestational age
dPN: postnatal day
dP/dtmax: the maximum first derivative
dP/dtmin: the minimum first derivative
Fr: French
IUGR: intrauterine growth restriction
KCl: potassium chloride
KH2PO4: potassium dihydrogen phosphate
LC/MS/MS: liquid chromatography-tandem mass spectrometry
LV: left ventricle
LVDP: left ventricular developed pressure
LVEDP: left ventricular end diastolic pressure
MIQE: minimum information for publication of quantitative real-time PCR experiments
MgSO_2_: magnesium sulfate
MitoQ: mitochondria-targeted antioxidant
NADPH: nicotinamide adenine dinucleotide phosphate
NaHCO_3_: sodium bicarbonate
Nfe2l2: nuclear factor, erythroid-like 2
NaCl: sodium chloride
RNA: ribonucleic acid
UPLC: ultra-high-performance liquid chromatography
