# Supplementary material for: Mitochondria antioxidant protection against cardiovascular dysfunction programmed by early-onset gestational hypoxia
Source: FASEB J. Author manuscript; Available in PMC 2021 Dec 8. (PMC7612077; doi:10.1096/fj.202002705R)
Supplement: Supporting information — Additional Supporting Information may be found online in the Supporting Information section. [file EMS140041-supplement-Supporting_information.pdf]

NeuroImage 237 (2021) 118175

Contents lists available at ScienceDirect

NeuroImage

journal homepage: www.elsevier.com/locate/neuroimage

Improved susceptibility weighted imaging at ultra-high field using bipolar multi-echo acquisition and optimized image processing: CLEAR-SWI

Korbinian Eckstein[a], Beata Bachrata[a,b], Gilbert Hangel[a,c], Georg Widhalm[c], Christian Enzinger[d], Markus Barth[e], Siegfried Trattnig[a,b], Simon Daniel Robinson[a,b,d,f,*]

[a] High Field MR Centre, Department of Biomedical Imaging and Image-guided Therapy, Medical University of Vienna, Vienna, Austria
[b] Karl Landsteiner Institute for Clinical Molecular MR in Musculoskeletal Imaging, Vienna, Austria
[c] Department of Neurosurgery, Medical University of Vienna, Vienna, Austria
[d] Department of Neurology, Medical University of Graz, Graz, Austria
[e] School of Information Technology and Electrical Engineering, Faculty of Engineering, Architecture and Information Technology, The University of Queensland, Brisbane, Australia
[f] Centre for Advanced Imaging, The University of Queensland, Brisbane, Australia

ARTICLE INFO

Keywords:
SWI
CLEAR-SWI
Ultra-high Field
Multi-echo
Brain tumor
T2*

ABSTRACT

Purpose: Susceptibility Weighted Imaging (SWI) has become established in the clinical investigation of stroke, microbleeds, tumor vascularization, calcification and iron deposition, but suffers from a number of shortcomings and artefacts. The goal of this study was to reduce the sensitivity of SWI to strong B_1 and B_0 inhomogeneities at ultra-high field to generate homogeneous images with increased contrast and free of common artefacts. All steps in SWI processing have been addressed – coil combination, phase unwrapping, image combination over echoes, phase filtering and homogeneity correction – and applied to an efficient bipolar multi-echo acquisition to substantially improve the quality of SWI.

Principal results: Our findings regarding the optimal individual processing steps lead us to propose a Contrast-weighted, Laplace-unwrapped, bipolar multi-Echo, ASPIRE-combined, homogeneous, improved Resolution SWI, or CLEAR-SWI. CLEAR-SWI was compared to two other multi-echo SWI methods and standard, single-echo SWI with the same acquisition time at 7 T in 10 healthy volunteers and with single-echo SWI in 13 patients with brain tumors. CLEAR-SWI had improved contrast-to-noise and homogeneity, reduced signal dropout and was not compromised by the artefacts which affected standard SWI in 10 out of 13 cases close to tumors (as assessed by expert raters), as well as generating T2* maps and phase images which can be used for Quantitative Susceptibility Mapping. In a comparison with other multi-echo SWI methods, CLEAR-SWI had the fewest artefacts, highest SNR and generally higher contrast-to-noise.

Major conclusions: CLEAR-SWI eliminates the artefacts common in standard, single-echo SWI, reduces signal dropouts and improves image homogeneity and contrast-to-noise. Applied clinically, in a study of brain tumor patients, CLEAR-SWI was free of the artefacts which affected standard, single-echo SWI.

1. Introduction

Susceptibility Weighted Imaging (SWI, Haacke and Reichenbach, 2011; Haacke et al., 2004; Reichenbach et al., 1997) is an MRI method which uses the sensitivity of the phase of the signal to magnetic susceptibility to generate contrast between tissues which contain different concentrations of calcium, deoxygenated blood, blood products and iron (Sehgal et al., 2005). This has led to clinical application in imaging pathologies including stroke (Santhosh et al., 2009), tumors (Grabner et al., 2017), vascular dementia (Ayaz et al., 2010) and multiple sclerosis (Absinta et al., 2016; Filippi et al., 2019; Rovira et al., 2015). SWI benefits from the sensitivity of high field (Reichenbach et al., 2000) and ultra-high field (Deistung et al., 2008; Koopmans et al., 2008) gradient echo imaging to susceptibility-induced variations in the static magnetic field, ΔB_0. At 7 T and higher field strengths, however, the simple complex filtering typically used to generate SWI, originally conceived for field strengths around 1.5 T, leads to gross artefacts in the presence of inhomogeneous B_1 and B_0 (Li et al., 2015).

In routine and research clinical use, SWI is still predominantly acquired using a single-echo gradient echo sequence and generated using homodyne high-pass filtering (Absinta et al., 2016; Bian et al., 2014; Dal-Bianco et al., 2015; Di Ieva et al., 2013; Frischer et al., 2012;

* Corresponding author.

https://doi.org/10.1016/j.neuroimage.2021.118175.
Received 8 March 2021; Received in revised form 28 April 2021; Accepted 13 May 2021
Available online 15 May 2021.
1053-8119/© 2021 The Authors. Published by Elsevier Inc. This is an open access article under the CC BY license (http://creativecommons.org/licenses/by/4.0/)

Moenninghoff et al., 2015; Ong and Stuckey, 2010; Schlamann et al., 2010; Tan et al., 2000; Theysohn et al., 2011). Acquiring multiple echoes in the same repetition time (TR) that 'Standard SWI' would require has a number of advantages, however. Early echoes capture signal from regions with short T_2*, and the multi-echo data acquired for SWI can additionally be used for a range of other methods, leading to improved QSM (Wang and Liu, 2015), providing a window into tissue microstructure (Wharton and Bowtell, 2013, 2012) and allowing T_2* relaxation to be mapped.

Combining phase information from the individual RF coils used in a phased array receiver coil is a crucial step in avoiding artefacts and obtaining optimum contrast from the phase (Robinson et al., 2017a). Homodyne filtering (Noll et al., 1991) eliminates low-frequency, coil-dependent contributions to the phase, allowing the phase data from different coils to be combined, but the process leaves wrap-like artefacts in regions of high ΔB_0 where the range of the filtered phase exceeds 2π (Li et al., 2015; Oh et al., 2014) as well as rendering the data unusable for Quantitative Susceptibility Mapping (QSM). Other phase combination approaches include the method of Roemer et al. (1990) and SENSE (Pruessmann et al., 1999), but these are restricted to systems with a homogeneous reference coil – i.e. up to 3 T. Multi-echo measurements open up alternative solutions, allowing the contributions to the phase which evolve over time and arise from ΔB_0 (and are of interest) to be separated from the 'phase offset' or 'initial phase' of each coil. A high CNR combined phase image is achieved by determining and removing smoothed or fitted approximations to the initial phase (Robinson et al., 2011). The ASPIRE method is a variant of this approach which avoids the need for phase unwrapping, making it computationally efficient and robust (Eckstein et al., 2018a).

Data from multi-echo acquisitions can be combined over echoes to reduce signal dropouts and maximize CNR (Luo et al., 2012). Magnitude data has been combined using simple averaging (Brainovich et al., 2009) and with root-sum-of-squares (RSS) to optimize SNR (Jutras et al., 2017). Phase data, on the other hand, has been combined by averaging the B_0 map derived from each phase image (Brainovich et al., 2009), which is improved by weighting B_0 maps (Quinn et al., 2014) but also by weighted least-squares fitting (Gilbert et al., 2012) or fitting a complex-valued mono-exponential signal decay (Luo et al., 2012).

Phase data need to be unwrapped and high-pass filtered prior to generating an SWI. In the standard approach, these two steps are subsumed by homodyne filtering. This leaves some wrap-like artefacts in regions of high ΔB_0 (Li et al., 2015; Oh et al., 2014), and it has been suggested that phase instead be unwrapped and high-pass filtered in separate steps (Oh et al., 2014; Rauscher et al., 2003; Schofield and Zhu, 2003), either using region growing (Rauscher et al., 2003; Schweser et al., 2011) or Laplacian unwrapping (Oh et al., 2014; Robinson et al., 2017a), the latter of which offers a faster and more robust solution.

The phase mask which provides the additional susceptibility in SWI is generated by scaling the unwrapped, filtered phase to the interval [0;1]. In Standard SWI, negative phase values (resulting from diamagnetic sources) have a filter value of 1 (i.e. no weighting) and positive phase values (from paramagnetic sources) are scaled linearly from 1 at the origin to 0 for the largest phase value present (Haacke et al., 2004). Quinn et al. (2014) have shown that noise can be reduced and contrast increased using a sigmoidal scaling function, indicating that the phase masking function is worthy of careful consideration.

At ultra-high field, inhomogeneous transmit and receive B_1 fields lead to intensity variations which make SWI hard to window and interpret. Homogeneity corrections used in other neuroimaging contexts require manual configuration (Unified Segmentation; Ashburner and Friston, 2005), have computation times not suited to online calculation (N3; Sled et al., 1998), require a homogeneous reference coil (Roemer et al., 1990) or are vendor specific and not publicly available. A fast and robust solution for gradient echo imaging at ultra-high field has been presented recently (Eckstein et al., 2018b), offering an alternative which is exploited here.

The aim of this study was to develop an improved multi-echo SWI without the problems which affect Standard SWI at ultra-high field; wrap-like artefacts in areas of high ΔB_0, signal dropouts and inhomogeneous appearance. The proposed approach, CLEAR-SWI (Contrast-weighted, Laplace-unwrapped, bipolar multi-Echo, ASPIRE-combined, homogeneous, improved Resolution SWI), employs the developments of multiple recent publications and newly devised steps to generate homogeneous SWI free of phase artefacts and with minimal signal dropout in regions with large field gradients. The magnitude data from the multi-echo scans can be used for T_2*/R_2*-mapping and the phase, processed as described, can be used for fitting multiple compartment models for microstructural analysis, frequency difference mapping and QSM. All the applied methods are considered with a view towards online implementation on the imaging hardware.

2. Theory

We begin by comparing the SNR achievable in single-echo acquisitions, multi-echo acquisitions in which signal is acquired under gradient lobes of the same polarity (monopolar) and those in which signal is acquired under both negative and positive gradient ramps (bipolar). The signal formulation is also the basis for calculating weighting factors for the contrast-weighted combination of multi-echo magnitude images proposed in a later section. In the SNR comparison, all sequence parameters were kept identical and only the sequence type (single-echo, monopolar, and bipolar), the number of echoes and the T_R were changed.

Assuming single-exponential T_2^* decay, the signal from one echo, S, is given by:

$$S = \rho \cdot f_\alpha\left(T_R, T_1, \alpha\right) \cdot f_{BW}\left(T_2^*, T_{acq}\right) \cdot e^{-\frac{T_E}{T_2^*}} \tag{1}$$

where ρ is the proton density, T_1 and T_2^* are longitudinal and transverse relaxation times, T_R the repetition time, T_{acq} the acquisition window, T_E the echo time, α the nominal flip angle, f_α the flip angle term, which is generally position-dependent, and is given by (Bernstein et al., 2004; p. 587);

$$f_\alpha\left(T_R, T_1, \alpha\right) = \frac{\left(1 - e^{-\frac{T_R}{T_1}}\right) \cdot \sin(\alpha)}{1 - e^{-\frac{T_R}{T_1}} \cdot \cos(\alpha)} \tag{2}$$

and the signal term f_{BW} describes signal averaging in the acquisition window of one echo T_{acq} – the inverse of the receiver bandwidth per pixel (Jutras et al., 2017);

$$f_{BW}\left(T_2^*, T_{acq}\right) = 2T_2^* \cdot \left(1 - e^{-\frac{T_{acq}}{2T_2^*}}\right) \tag{3}$$

This term grows linearly for acquisition windows which are short compared to T_2^*, then flattens out when increasing the acquisition window.

As the simulated SNR is considered only in relative terms, with changing T_{acq}, the noise is assumed to be independent over time and thus proportional to $\sqrt{T_{acq}}$, leading to the SNR formulation

$$\mathrm{SNR} \propto \frac{S}{\sqrt{T_{acq}}} \tag{4}$$

To calculate the SNR of a combined image from a multi-echo measurement, the close-to-optimum SNR method RSS is used on the signal from echoes, with the SNR being (Jutras et al., 2017)

$$\mathrm{SNR}_{ME} = \sqrt{\sum \mathrm{SNR}_i^2} \tag{5}$$

where SNR_i is the SNR of the i-th echo.

SNR efficiency (SNRe; Sodickson et al., 1999) is generally used to compare the SNR of acquisitions with different total acquisition time, which is proportional to the T_R, and is given by

$$\mathrm{SNRe} \propto \frac{\mathrm{SNR}}{\sqrt{T_R}} \tag{6}$$

3. Materials and methods

3.1. Measurements

Measurements were performed on volunteers and patients. To systematically compare Standard single-echo SWI with multi-echo CLEAR-SWI, acquisitions with identical nominal resolution, repetition time (TR) and GRAPPA factor – and thus identical measurement time – were carried out. An overview of the measurements can be found in Table 1 and specific considerations in the next paragraphs.

3.1.1. Scanners and participants

Measurements with 10 healthy subjects (3F, 7M, 31.4 ± 6.7 years old) and 14 patients with suspected brain tumors (7F, 7M, 52.6 ± 15.2 years old) were performed using a 7 T MRI Siemens MAGNETOM scanner (Siemens Healthineers, Erlangen, DE) with a 32-channel Nova Medical head coil (Wilmington, Massachusetts, USA). One patient was diagnosed with CLIPPERS and removed from the cohort. The diagnoses of the patients with brain tumors included astrocytoma WHO grade II/III, glioblastoma multiforme WHO grade IV, meningioma WHO grade I, and brain metastasis of an adenocarcinoma (details can be found in Supplementary Information Appendix H). The measurements were approved by the Ethics Committee of the Medical University of Vienna and all participants provided written informed consent.

3.1.2. Volunteer measurements

The same resolution, TR, parallel imaging (GRAPPA) acceleration factor and acquisition time was used for the single and multi-echo protocols in the volunteer study, but with different bandwidths and number of echoes. The protocols were based on recent clinical 7T SWI studies in which a TE of 20 ms was used (Najdenovska et al., 2019; Pittau et al., 2018), but with resolution, and thus the measurement time, increased. All acquisitions were 3D gradient echo scans with Partial Fourier 6/8 in both phase and slice direction.

The single-echo volunteer scan (see Table 1; *NE1*) was acquired with a bandwidth of 60 Hz/pixel and TE = 19.3 ms to use as much as possible of the TR for sampling, to ensure a fair comparison with the multi-echo acquisition. Parameters in the multi-echo acquisition, *NE6*, were selected to efficiently use the same TR as the single-echo scan while enabling phase data to be combined over channels using ASPIRE, for which there is the requirement, for bipolar acquisitions, that $2T_{E,1} = T_{E,2}$ and $3T_{E,1} = T_{E,3}$. Fig. 4 (Simulation Results) was used as a guideline for choosing the number of echoes in the bipolar acquisition. The resulting acquisition parameters are similar to what was proposed by Liu et al. (2017) as 5-echo SWI for 7 T, but with one additional echo and therefore a slightly longer TR.

The measurements used in the SNR comparison were made after an upgrade of the MRI scanner from 7T MAGNETOM to 7T Plus, which entailed an update of control software and sequences, and with that, the need to make minor modifications to protocols (*NE1* cf. *NE1 SNR* and *NE6* cf. *NE6 SNR* in Table 1). The scans *NE1 SNR* and *NE6 SNR* were each repeated 3 times. From the 3 repeated measurements, displacements were estimated using FSL FLIRT (Jenkinson et al., 2002) and the pair with least displacement was used for calculating SNR values.

3.1.3. Tumor measurements

For reasons of patient tolerance, the duration of the CLEAR-SWI tumor scan was decreased to 5 min 10 s by increasing the GRAPPA factor (compared to the *NE6* scan) from 2 to 3. The number of echoes (10) and bandwidth (523 Hz/pixel) for the tumor scan were chosen prior to the development of the SNR model presented here, and are estimated to yield 20% lower SNR than the optimum number of echoes (6) and bandwidth (243 Hz/pixel) (compare Fig. 4). In patients, it was not possible to acquire a Standard SWI in addition to CLEAR-SWI due to measurement time restrictions. For this reason, and to ensure consistency in scan-dependent factors such as head position and motion,

Table 1
Parameters of the acquisitions. The multi-echo scans were all acquired using a bipolar readout.

Scan	TR [ms]	NE	TE [ms]	FA [°]	BW [Hz/px]	Matrix	Res [mm^3]	FOV	GRAPPA	Time
NE1	30	1	19.3	15	60	800 × 600 × 104	0.26 × 0.26 × 1.2	210 × 158 × 125	2	9 min 35 s
NE6	30	6	[4.30,8.60,12.90,17.20,21.50,25.80]	15	260	800 × 600 × 104	0.26 × 0.26 × 1.2	210 × 158 × 125	2	9 min 35 s
NE1 SNR	30	1	20.0	10	80	800 × 600 × 104	0.28 × 0.28 × 1.2	224 × 168 × 125	2	9 min 35 s
NE6 SNR	30	6	[4.10,8.20,12.30,16.40,20.50,24.60]	10	270	800 × 600 × 104	0.28 × 0.28 × 1.2	224 × 168 × 125	2	9 min 35 s
Tumor	31	10	[3.31,6.62,9.93,12.49,15.05,17.61,20.17,22.73,25.29,27.85]	10	523	736 × 552 × 80	0.30 × 0.30 × 1.2	220 × 164 × 96	3	5 min 10 s

simulated Standard SWI were generated for tumor patients from multi-echo CLEAR-SWI data, as described in Section 3.4.

3.2. Analysis

The Siemens 7T MAGNETOM VB17 implementation of SWI was used for Standard SWI. Images from Standard SWI and CLEAR-SWI were compared quantitively (Section 3.3) and qualitatively. In the qualitative assessment, emphasis was placed on the level of detail visible, intensity homogeneity and the presence of artefacts – primarily signal dropout and unresolved wraps. The computation time of CLEAR-SWI was measured on a system with 32 Gb memory and an Intel® Core^{TM} i7-7700 processor running Ubuntu 16.04.

3.3. SNR and CNR assessment

A measurement of SNR and CNR was performed for magnitude images on manually defined pairs of ROIs, where each pair included one ROI in white matter and one in a second tissue; cortical grey matter (GM), globus pallidus (GP), the optic radiation (OR), red nucleus (RN), substantia nigra (SN). To avoid a biased selection of ROIs, the ROI pairs were defined on the single-echo magnitude multiplied by the sixth echo of the multi-echo magnitude. The ROIs were spherical, spanned 3 slices and comprised 111 voxels. The cortical GM ROI pairs were distributed over slices spanning the full (head-foot) extent of the brain. The total number of ROI pairs was 261 (87 GM, 48 GP, 64 OR, 26 RN, 36 SN).

The SNR and CNR values were calculated from two repeated scans, where for each region, the signal was calculated as the mean value in both scans and the noise as the standard deviation of the difference between the two scans divided by $\sqrt{2}$ (NEMA method 1; Goerner and Clarke, 2011). SNR was calculated as signal divided by noise for each ROI individually and CNR as the difference in signal of an ROI pair divided by the mean noise in both ROIs.

3.4. SNR simulation

Echo times $T_{E,i}$, sampling windows T_{acq} and repetition times T_{R} were calculated for single-echo, and monopolar and bipolar multi-echo sequences for a range of bandwidths. For multi-echo acquisitions, the bandwidth was kept constant for all echoes. The echo times and sampling windows were chosen to maximize the total sampling time for the given T_{R}, while using equally spaced echoes that fulfilled $T_{E,i} = i \cdot \Delta T_E$ in the multi-echo case. This calculation is described in more detail in Supplementary Information, Section A, including a consideration of gradient ramps and prewinder gradients. The parameters used for the simulation were 7 T field strength, 200 T/m/s slew rate and 42 mT/m maximum gradient strength in readout direction, corresponding to the parameters of the SC72 gradients, the most common gradient set on Siemens 7 T systems. The gradient parameters were obtained from sequence simulations generated using the Siemens tool POET with gradient mode “fast”. The time required for excitation was 2.1 ms and 3 ms for spoiling.

The duty cycle calculation was performed per echo for a given echo spacing and multiple matrix sizes in the readout direction. It was calculated as

$$D = T_{\mathrm{acq}} / \Delta T_E \qquad (7)$$

SNR efficiency was calculated for each simulated acquisition ($T_{E,i}$, T_{acq}, T_{R}) using the SNR theory described in Section 2 with the considered tissue being white matter with $T_2{}^*$ of 26.8 ms (Peters et al., 2007) and T_1 of 1126 ms (Wright et al., 2008).

3.5. Standard SWI reference from multi-echo data

For comparison between CLEAR-SWI and Standard SWI in cases where no single-echo data was acquired (*Tumor)*, Standard SWI was generated from the multi-echo acquisition. The phase and magnitude were calculated separately from the multi-echo data, reproducing the signal characteristics very similar to those that would have been measured in a single-echo scan, whilst ensuring that slice positioning and motion were identical to that in CLEAR-SWI.

The magnitude was a weighted combination of the multi-echo magnitude, using echo-dependent weighting factors w_i, which were equal to the fraction of the echo that lies inside the acquisition window of the simulated single-echo scan.

The simulated phase was generated by unwrapping the multi-echo phase using ROMEO (Dymerska et al., 2021) prior to combination according to:

$$\theta = T_E \frac{\sum \theta_i w_i}{\sum T_{E,i} w_i} \qquad (8)$$

where the weighting factors w_i are the same as those used for the magnitude, T_E is the echo time of the Standard SWI and θ_i are the unwrapped multi-echo phases with echo times $T_{E,i}$.

The phase θ was wrapped into the range $[-\pi; \pi)$ and the default SWI processing steps of homodyne filtering, phase mask creation and 4 times multiplication onto the magnitude were performed (Haacke et al., 2004). The Standard SWI reference from multi-echo data was affected by the same artefacts as single-echo Standard SWI (see Supplementary Information, Section B).

3.6. Expert rating

A neuroradiologist (ST), a neurosurgeon (GW) and a neurologist (CE), each with over 20 years of experience, were asked to independently rate SWI images from the 13 tumor patients. For each patient, Standard SWI and CLEAR-SWI were presented next to each other, unlabeled, with random attribution of which image was on the left and the right. Images were viewed with MRIcro (https://people.cas.sc.edu/rorden/mricro/mricro.html) in synced scrolling mode. Raters could freely scroll through the slices and were tasked to rate 4 different aspects: the *Visibility of the Pathology* (considering boundary, vasculature, internal structure, blood breakdown products, other features of interest in assessing the pathologies) and *Overall Quality* (estimate the overall quality) on a scale from 1 (lowest quality) to 6 (highest quality); the *Artefacts in and around the Pathology* (considering wrap artefacts, signal dropouts and other degrading artefacts impeding analysis of the pathology) and *Overall Artefacts* (considering inhomogeneity, wrap artefacts, signal dropouts, and other artefacts that were visible) on a scale from 1 (no artefacts) to 6 (severe artefacts).

3.7. Multi-Echo SWI methods for comparison

CLEAR-SWI was compared to two advanced multi-echo SWI methods, GEPCI (Luo et al., 2012) and that proposed by Quinn et al., (2014). The SWI method of Quinn et al., which was developed for 3 T, was implemented according to the steps described in the publication other than that the homodyne filter size was reduced from 30% of the FOV to 3T/7T * 30% FOV = 13% FOV. The filter size was reduced to adjust for the stronger expected background field, although this only slightly mitigated the homodyne filtering artefacts. GEPCI was implemented as described in the publication (Luo et al., 2012), with details regarding complex fitting in the Supplementary Information, Appendix E.

3.8. Availability of code and data

RF coil signals were combined on the scanner image reconstructor using ASPIRE, which is available from the authors as a C2P package for VB17, VE11 and VE12, and as a MATLAB repository[1]. The CLEAR-SWI

[1] https://github.com/korbinian90/ASPIRE

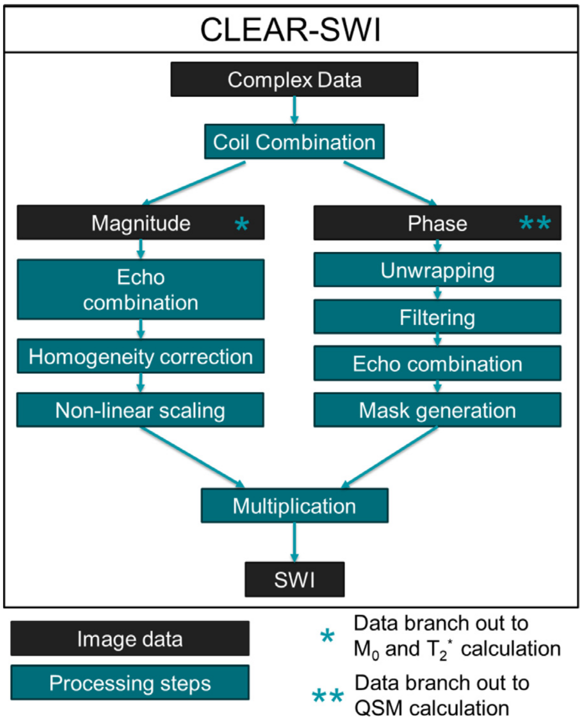

Fig. 1. Proposed SWI processing pipeline; The black boxes indicate images and the dark green boxes indicate processing steps in CLEAR-SWI.

image processing pipeline was implemented in the open source language Julia (Bezanson et al., 2017) and is available on GitHub[2]. The homogeneity correction can be accessed separately on GitHub[3]. The code used for the simulation of duty cycle and SNR is also available on GitHub[4]. Data from the 5 healthy subjects included in the quantitative comparison is available on the Harvard Dataverse (Eckstein et al., 2021). In the absence of an explicit and dedicated consent, patient data have not been made available.

3.9. CLEAR-SWI Preprocessing steps

The proposed SWI processing pipeline (Fig. 1) comprises coil combination of complex data; echo combination and homogeneity correction of magnitude images; unwrapping, filtering, echo combination, mask generation of phase images; and final multiplication of the processed magnitude and phase image.

3.9.1. Coil combination

The choice of the coil combination algorithm is not only crucial in order to avoid artefacts such as open-ended fringe lines, but also to achieve high magnitude SNR and phase CNR (Robinson et al., 2017a). Several methods exist which avoid phase artefacts, although many are subject to particular restrictions. The Roemer/ SENSE (Roemer et al., 1990) approach requires a reference coil, which limits its applicability at ultra-high field strengths and parallel transmit (PTx) coils. The phase difference method (also called Hermitian inner product) works for all multi-echo sequences but suffers from markedly reduced phase CNR. The VRC approach (Parker et al., 2014) would be applicable in general and works for single-echo data but needs to detect a valid center for the phase correction, which is a fragile, image-dependent step, and – dependent on the coil geometry – can generate artefacts above 7 T, or at 7 T for objects larger than the head (Robinson et al., 2017a). For multi-echo data, the ASPIRE method of coil combination (Eckstein et al., 2018a) meets all the requirements of this study; it does not require a reference coil, making it suitable for all field strengths and PTx coils, it is not dependent on image-derived parameters and the CNR is higher than in phase difference methods. The only limitation of ASPIRE is that two echoes satisfy $(m+1)T_{E,1} = mT_{E,2} | \; m \in \mathbb{N}$ for monopolar acquisitions and, for bipolar acquisitions, that three echoes satisfy $2T_{E,1} = T_{E,2}$ and $3T_{E,1} = T_{E,3}$.

The effect of the coil combination method on the magnitude images is less crucial, although, due to the Rician noise distribution in magnitude images, the classic root-sum-of-squares combination incurs a bias, which is stronger for areas with low intensity (e.g. veins). ASPIRE reduces this effect by performing magnitude weighted combination of the complex data after the removal of coil specific phase offsets.

3.9.2. Noise mask generation

A brain mask was required for high-pass filtering the phase to avoid image background affecting the values in voxels at the border of the brain. A conservative mask was created by thresholding the image at the value $\mu_{noise} + 2\sigma_{noise}$, where μ_{noise} and σ_{noise} are estimates of the noise mean and standard deviation respectively. For the sequence type used and for imaging brains, the background of the image contains mostly noise. Out of the eight corners of the 3D image matrix, that with the lowest mean value in a 10 × 10 × 10 volume was identified and the noise mean and standard deviation were calculated from that volume. The noise level estimation might need modification if applied to other sequences.

3.10. CLEAR-SWI phase processing steps

3.10.1. Phase unwrapping

Phase images were unwrapped using Laplacian unwrapping Schofield and Zhu (2003), a faster and more robust method than path-based or region-growing spatial unwrapping. The arbitrary background field variations introduced by this process were removed by subsequent high-pass filtering, allowing unwrapped phase data to be combined over echoes without bias.

3.10.2. Phase high-pass filtering

A masked, Gaussian low-pass filter with a sigma of 4 pixels was applied to the unwrapped phase and the resulting image subtracted from the original image to achieve high-pass filtering. The mask from step 3.9.2 was used to avoid the inclusion of noise voxels. Filtering was applied slice-wise, which provides a satisfactory result in terms of CNR, for SWI data, which typically have an aspect ratio of 2 or more (Deistung et al., 2008). For the more isotropic voxels commonly acquired for QSM, 3D high-pass filtering might yield higher phase CNR. A comparison of 2D vs 3D filtering and different kernel sizes is presented in Supplementary Information, Section C.

3.10.3. Multi-echo combination of phase

The unwrapped and high-pass filtered phase images from multiple echoes were combined using SNR-optimal inverse-variance-weighted frequency averaging (Quinn et al., 2014). The frequency is calculated as $\varphi_i / T_{E,i}$. With the voxelwise weights

$$w_i = T_{E,i}^2 M_i^2 \tag{9}$$

this leads to the combined phase

$$\varphi = \frac{\sum w_i \varphi_i / T_{E,i}}{\sum w_i} \tag{10}$$

[2] https://github.com/korbinian90/ClearSwi.jl
[3] https://github.com/korbinian90/MriResearchTools.jl
[4] https://github.com/korbinian90/MriResearchTools.jl/tree/clearswi_publication

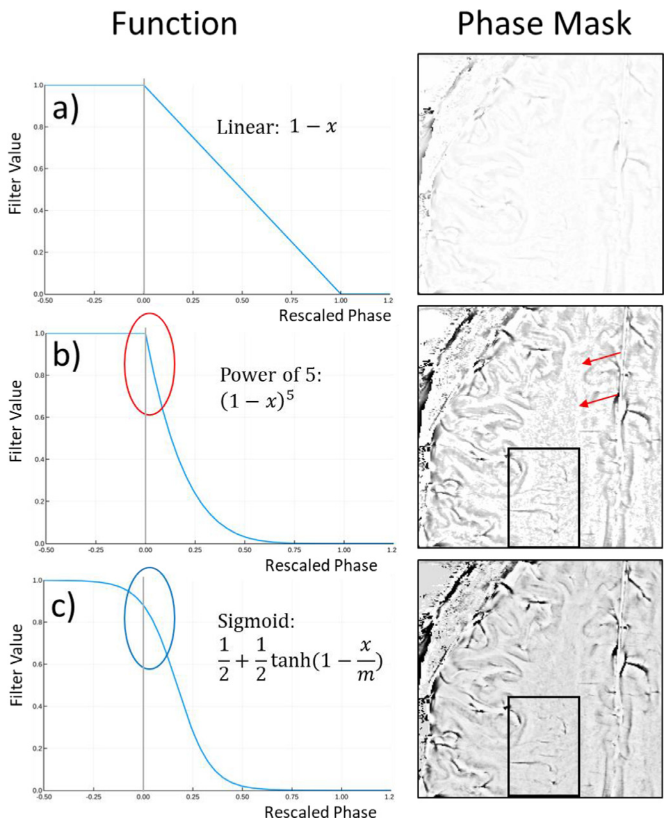

Fig. 2. A comparison of phase masking functions described in the text. The linear function a) yields low weighting in the phase mask. A power function with an exponent of 5 (equivalent to 5 applications of the linear function a)) results in the desired phase contrast in veins and grey matter but strongly amplifies noise (red arrows), because of the steep part of the function close to 0 (red ellipse). The sigmoid function c) (with level=4, resulting in m=0.17) is less steep close to the origin (blue ellipse), leading to lower noise in the phase mask, but achieves similar weighting in veins and grey matter (black box).

3.10.4. Sigmoidal phase masking

The phase mask used in SWI is conventionally generated with the positive, linear function

$$f(x) = \begin{cases} 1, & \text{if } x < 0 \\ 1 - x, & \text{if } 0 \le x \le 1 \\ 0, & \text{if } x > 1 \end{cases} \tag{11}$$

which is applied voxelwise to the high-pass filtered phase. To achieve the desired phase weighting in the final SWI, the magnitude is multiplied by the phase mask k times. This is equivalent to a single multiplication of the phase mask generated with the following power function:

$$f_k(x) = \begin{cases} 1, & \text{if } x < 0 \\ (1 - x)^k, & \text{if } 0 \le x \le 1 \\ 0, & \text{if } x > 1 \end{cases} \tag{12}$$

Fig. 2 illustrates the fact that higher power (k) amplifies noise, which has values close to 0 (Fig. 2b). To avoid noise amplification around 0 but still achieve strong phase contrast, the weighting function should be flat around 0 and steep thereafter. This behavior is provided by sigmoidal functions, such as tanh, which we adopt here (Fig. 2c), with a further comparison of standard and sigmoidal phase masking in Sup. Fig. C-1. As a reference for scaling, the median of the positive phase values was used with an additional *level* parameter for adjusting the intensity of the phase mask:

$$m = \text{median}_{>0}(\varphi) \cdot \text{level} \tag{13}$$

$$f_{\tanh}(x) = \frac{1}{2} + \frac{1}{2}\tanh\left(1 - \frac{x}{m}\right) \tag{14}$$

3.11. CLEAR-SWI magnitude processing steps

3.11.1. Multi-echo magnitude combination

We outline two alternative weighted combinations of magnitude images in CLEAR-SWI: SNR-weighted and CNR-weighted.

Optimum SNR in the combined magnitude image can be achieved by root-sum-of-squares weighting over echoes (Wu et al., 2012a), as (assuming the same bandwidth for each echo, and thereby the same noise) weighting by the magnitude corresponds to weighting by SNR. This combination lessens the impact of signal dropouts by weighting early echoes higher in regions with rapid T_2^* decay.

If the distinction between two tissue types is of interest, the contrast can be optimized using multi-echo information. The weighting factors which optimize the CNR between two tissues can be calculated given the corresponding T_1, T_2^* and ρ values. We take the example of white matter (WM) and grey matter (GM), but the same process holds for e.g. WM and veins, or WM and WM-lesions. Optimum CNR in combined images is achieved by weighting each echo by the CNR for that echo, or (given that noise is constant across echoes), the contrast for each echo, C_i. The combination and weighting factors are given by

$$M = \frac{\sum w_i M_i}{\sum w_i} \tag{15}$$

$$w_i = C_i = S(\text{gm}, \text{T}_{E,i}) - S(\text{wm}, \text{T}_{E,i}) \tag{16}$$

with S being the signal formulation (Eq. 1, Theory) and w_i is a weight that is identical for each voxel. If the flip angle α varies strongly in space, a flip angle map can be used instead of a scalar value in the calculation of S, however, this requires an additional measurement and was not further investigated in this study.

If tissue parameters are not known, a relative intensity term R (related to the mean signals in ROIs in the two tissue types of interest, A_1 and A_2, obtained in M_0; see Section 3.12) and T_2^* can be estimated from the scan of interest or an existing scan with the same protocol.

$$R = \overline{M_0(A_2)} / \overline{M_0(A_1)} \tag{17}$$

$$w_i = e^{\frac{T_{E,i}}{T^*_{2,1}}} - Re^{\frac{T_{E,i}}{T^*_{2,2}}} \tag{18}$$

3.11.2. Magnitude homogeneity correction

At field strengths above 3 T, the combined magnitude image from a multi-channel array has high signal variations due to inhomogeneous transmit and receive B_1 fields. The signal variation can be expressed as a multiplicative bias field, which is identical for multi-echo and single-echo acquisitions and also for individual and combined echoes. We propose a new, simple and robust homogeneity correction in which the bias field is estimated, smoothed and removed without affecting CNR.

The first step in the homogeneity correction is a simple segmentation of the magnitude image from the first echo; this has the lowest tissue contrast and is least affected by signal dropouts. It is assumed that white matter is present over the whole brain, and that inside a small box (1/15 of the edge lengths of the FOV), the intensity of white matter is constant and can be detected at the 90% quantile of the intensity values. The segmentation roughly isolates white matter by removing all voxels inside the box that deviate by more than 10% from the detected white matter intensity. This is performed in an overlapping box-wise approach conducted in 3D with an overlap of half the box size in all 3 dimensions, so each voxel is contained in 8 boxes and is classified as being white matter if it meets the 'less than 10% deviation' criterion in at least 2 of the 8 boxes. Removal of too many white matter voxels or the inclusion of some voxels of a different tissue type only has a modest influence on the estimated bias field (Eckstein et al., 2018b).

To obtain a smooth bias field from the segmented image, a modified moving-window average filter is applied along each direction of the image matrix independently; a process which is repeated 4 times. The average filter is modified to interpolate and extrapolate if missing values are encountered. The iterative application of the average filter in all dimensions corresponds to 3D Gaussian smoothing with a sigma that can be chosen through adapting the filter sizes of the average filter. The combined magnitude image was divided by the bias field to remove inhomogeneity.

3.11.3. Magnitude non-linear scaling

Although the combined, homogeneous magnitude has higher CNR than Standard SWI, the contrast between deep grey matter structures and surrounding white matter is often weaker. This is caused by the high weighting attributed to early echoes and can be addressed by non-linear grey scaling of the image, without sacrificing CNR. As the magnitude values inside the brain after homogeneity correction are mostly in the upper half of the brightness scale, the range assigned to the lower half can be reduced, and that to the upper half expanded, effectively increasing all relevant contrasts. To keep the appearance natural, this is performed by applying the softplus function

$$f(x) = \frac{\log\left(1 + \exp\left(a(x - b)\right)\right)}{a} \tag{19}$$

The cutoff parameter b was set to half the 0.8-quantile and the softness parameter $a = 2$ was used, which resulted in magnitude contrast in similar strength to standard single-echo (see Sup. Fig. E-1).

3.12. Additional processing steps

A 2-parameter least squares fit to the ASPIRE combined multi-echo magnitude images was performed with the function

$$f\left(M_0, T_2^*\right) = M_0 e^{-T_E/T_2^*} \tag{20}$$

where T_E is the echo time, T_2^* the exponential decay constant of the tissue over echo time and M_0 the magnitude at time 0 after the excitation. M_0 is not affected by T_2^* decay, but contains proton density and T_1 weighting. T_2^* calculation was also performed online using the fast numeric method NumART_2^* (Hagberg et al., 2002). M_0 and T_2^* images for patients with brain tumors are shown in Fig. 11 and Supplementary Information, Section H.

4. Results

The results are organized into four sections: 4.1 Simulation, presenting SNR calculations for monopolar and bipolar multi-echo acquisitions; 4.2 Acquisition and preprocessing, comparing magnitude images from weighted magnitude-combined bipolar acquisitions with those from single-echo acquisitions; 4.3 SWI processing, presenting phase processing, homogeneity correction and the resulting CLEAR-SWI images and 4.4 Application with brain tumors, showing examples from patients with suspected brain tumors.

4.1. Simulation

4.1.1. SNR simulation

The sampling duty cycles for monopolar and bipolar acquisitions are given in Fig. 3 for a range of readout matrix sizes. The duty cycle of a single-echo acquisition would be always close to 100% and is not shown. Monopolar acquisitions had significantly lower duty cycle, with a maximum readout length of 512 pixels given the parameters and system constraints, while bipolar acquisitions were possible with resolutions of 960 and above, with duty cycles consistently in excess of 90%.

Predictions of SNR efficiency are shown in Fig. 4. Bipolar acquisitions achieved significantly higher SNRe than single-echo, other than for very short TR (< 15 ms). Monopolar acquisitions had consistently lower SNRe than bipolar acquisitions due to shorter T_{acq} periods.

Number of Pixels in Readout Direction		64	128	256	512	768	960
Duty Cycle	Monopolar	90%	85%	75%	51%	--	--
	Bipolar	99.6%	99.1%	98%	96%	94%	93%

Fig. 3. A comparison of the duty cycles of monopolar and bipolar acquisitions for a range of readout matrix sizes. Monopolar acquisitions had lower duty cycles due to the time spent executing rewinder gradients, and thereby lower predicted SNR. With the echo spacings used in the simulation, the largest readout matrix sizes were only possible with bipolar acquisitions. The echo spacing for the simulation was 4 ms, the field strength 7 T, the slew rate 200 T/m/s, the maximum gradient strength 42 mT/m and the FOV 210 mm.

4.1.2. Standard SWI from multi-echo data

Tests in a healthy subject, from whom both a multi-echo and single-echo scan were acquired, confirm that the simulated single-echo magnitude and phase, generated from the multi-echo scan (the approach used to generating single-echo data for the brain tumor patient group) corresponded closely to the single-echo scan (see Sup. Fig. B-1). The quality of the calculated single-echo SWI was also comparable to the acquired Standard SWI and was similarly affected by artefacts, both in severity and location.

4.2. Acquisition and preprocessing

The magnitude image from a multi-echo scan with CLEAR SNR-weighting over echoes (RSS combination) showed less signal dropout in regions with strong ΔB_0 gradient than the Standard, single-echo magnitude (Fig. 5, at red arrows). Further comparisons of magnitude signal dropout against other multi-echo SWI methods are presented in Supplementary Information, Figure E-1, which shows stronger signal dropouts in GEPCI and single-echo, while magnitude averaging (Quinn et al., 2014), like CLEAR-SWI, recovers most of the signal dropouts from early echo information.

4.3. SWI processing

4.3.1. SWI processing steps

Standard homodyne phase filtering (7T MAGNETOM VB17 implementation) is compared with the CLEAR steps of Laplacian unwrapping, Gaussian high-pass filtering and combination over echoes in Fig. 6. Wrap-like artefacts are present in the homodyne-filtered phase only (at red arrows).

The SNR and CNR of magnitude images from single-echo acquisitions and multi-echo acquisitions using a number of approaches to echo combination are compared in Table 2. SNR-weighting, CNR-weighting and magnitude averaging (used by Quinn et al.) provide magnitude images with higher SNR than GEPCI and single-echo magnitudes.

The CNR evaluation presented in Fig. 7 shows that the performance of each method depends on the tissue. CNR-weighting (optimized with literature values for WM and GM) and averaging (Quinn et al.) provide consistently high CNR values. SNR-weighting and GEPCI have significantly lower CNR in some regions and single-echo has lowest CNR in all regions (significance tests in Supplementary Information, Section E).

The effectiveness of the magnitude homogeneity correction is illustrated in triplanar view in Fig. 8. Without homogeneity correction, multi-echo magnitude images combined with CNR weighting show high signal close to coil elements and reduced brightness in the center (Fig. 8, left). The homogeneity correction has been applied to the same data (Fig. 8, right), removing non-anatomical signal variation.

4.3.2. SWI comparison

Standard SWI (*NE1*) is compared with CLEAR-SWI (*NE6*) in Fig. 9 and Fig. 10. Standard SWI contained a “worm-hole” artefact (Fig. 9 at

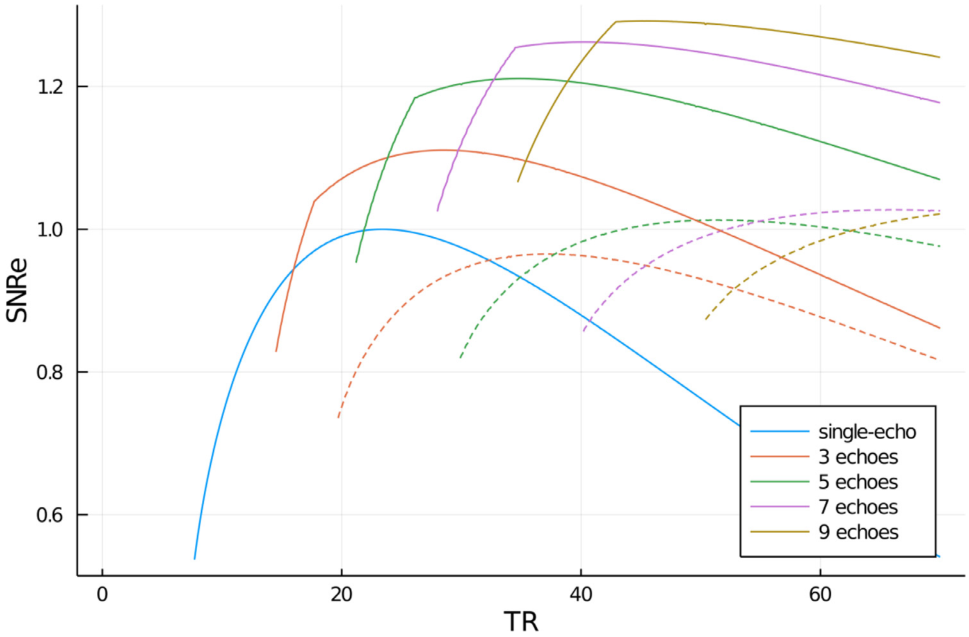

Fig. 4. Calculated SNRe in single-echo and multi-echo 3D GRE scans at 7 T. Bipolar acquisitions are illustrated with solid lines and monopolar acquisitions with dashed lines. The SNRe is calculated for an RSS combination over echoes. The matrix size in the readout direction was 800 pixels, the FOV 210 mm, T_{exc} was 2.1 ms, T_{spoil} was 3 ms. Results are for white matter with $T_2{}^*$ of 26.8 ms and T_1 of 1126 ms. Echoes were equally spaced and had $T_{E,2} = 2T_{E,1}$. Calculations for acquisitions with an even number of echoes are not shown to avoid overcrowding the figure. Note that the beginning of the bipolar lines is very steep, which is due to the restriction to use equidistant echoes.

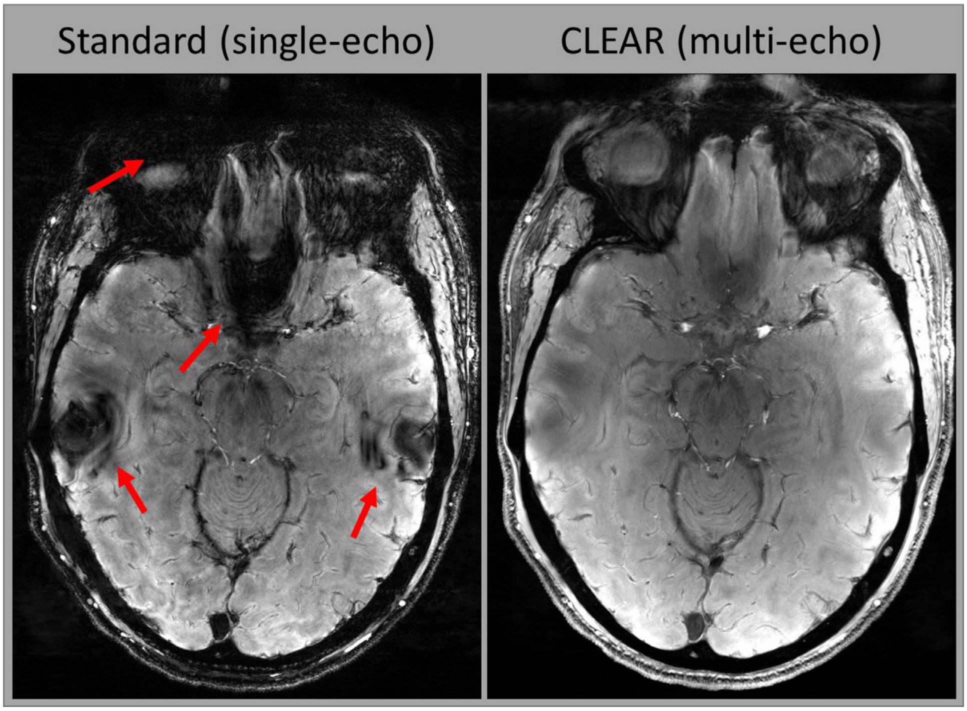

Fig. 5. A comparison of signal loss in the magnitude images from a Standard, single-echo acquisition and a multi-echo with CLEAR combination with SNR weighting; severe signal dropouts are present in single-echo in the slice illustrated (red arrows). The multi-echo scan has only minimal signal dropout in the same areas. The two magnitude images are from scans *NE1* and *NE6* combined over the echoes using SNR weighting (RSS). Intensity variations have not been corrected and are the same in both acquisitions.

Table 2
Average SNR across all ROIs from 5 volunteers. The SNR of both CLEAR combinations and magnitude averaging (Quinn et al.) is about twice the SNR achieved with GEPCI or single-echo acquisition.

Combination Method	CLEAR SNR-weighted	CLEAR CNR-weighted	Quinn et al.	GEPCI	Single-echo
SNR	35.5	30.7	32.4	17.3	15.4

red arrows) and wrap-like artefacts (Fig. 10 at red arrows) not present in CLEAR-SWI. Veins were more distinct in CLEAR-SWI, which were also less noisy (Fig. 9 and Fig. 10 - red boxes). Inhomogeneities in Standard SWI (Fig. 9 and Fig. 10 - blue arrows) were eliminated by the homogeneity correction in CLEAR-SWI. Fig. E-2 in Supplementary Material compares the visibility of veins in CLEAR-SWI with GEPCI and the SWI from Quinn et al. In general, veins were slightly better resolved in CLEAR-SWI. In certain regions (shown in the upper part of E-2), GEPCI and Quinn et al. had severely reduced vein visibility. An extended image comparison between Standard SWI and CLEAR-SWI of veins and 3 different slice locations with 5 volunteers can be found in Supplementary Information, Section G.

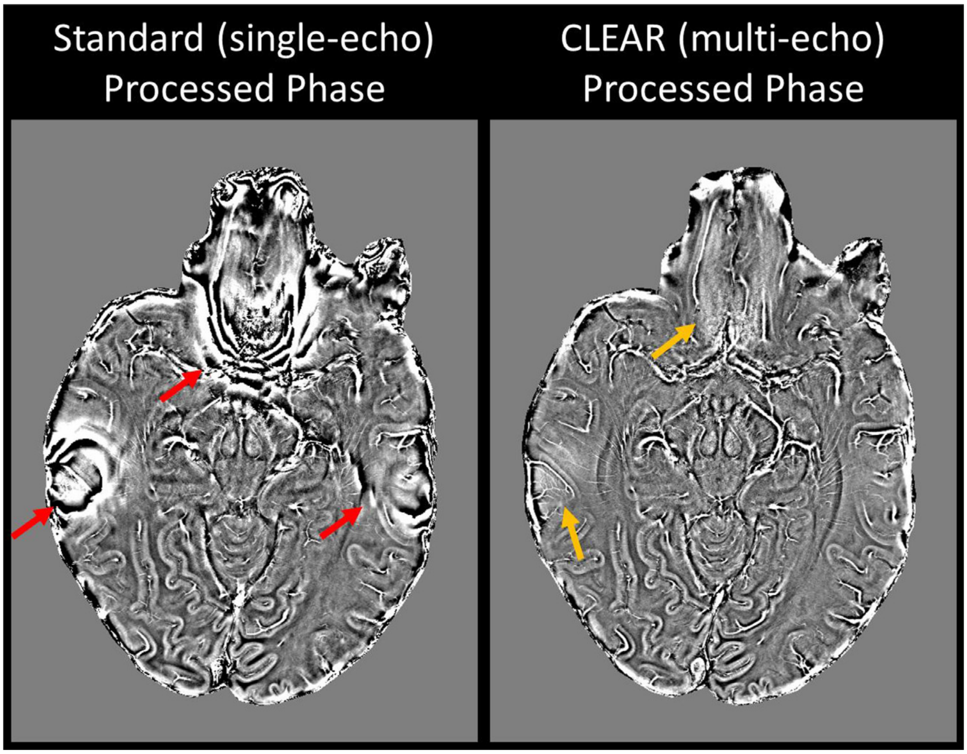

Fig. 6. The Standard homodyne-filtered single-echo image (left) shows wrap-like artefacts in regions with high ΔB_0 (red arrows) which are not present in the CLEAR Laplacian-unwrapped multi-echo phase (right). There is only a small residual signal variation in these regions in the CLEAR images (orange arrows). That could be removed using a smaller σ size in high-pass filtering at the expense of an increase in image noise.

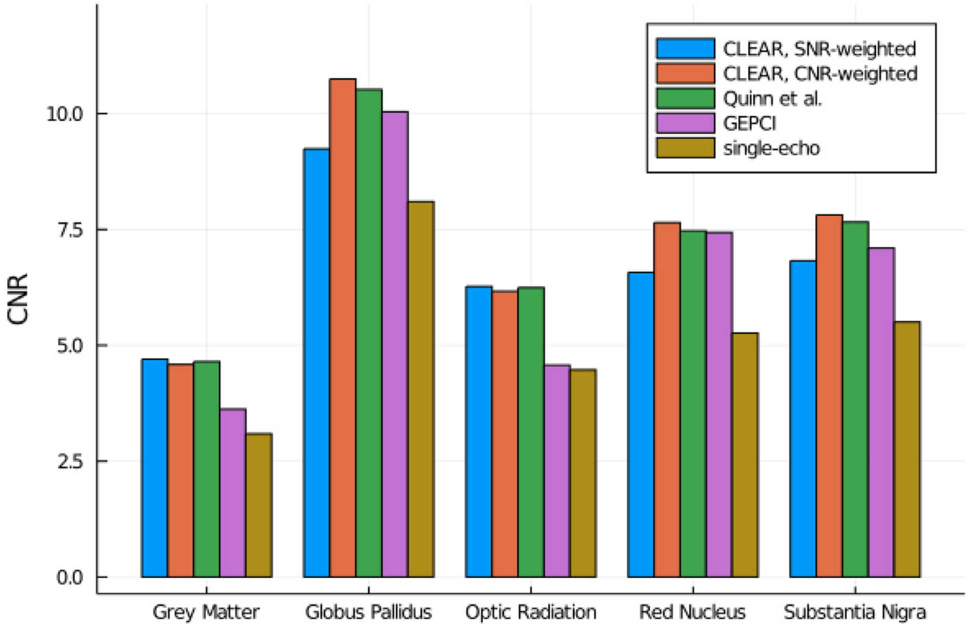

Fig. 7. CNR comparison between the different magnitude combination methods. CNR-weighting was performed between WM and GM using literature values. Most clearly visible differences are also statistically significant (the results of all Wilcoxon sign rank tests are shown in Supplementary Information Appendix E, Table E-1).

4.3.3. Minimum intensity projection

Minimum intensity projection images from Standard SWI (*NE1*) and CLEAR-SWI (*NE6*) illustrate the absence of wrap-like artefacts in the proposed method, which otherwise dominated the lower slices (Fig. 11). Fine structures were better resolved in CLEAR-SWI (compare red arrows), which were also more homogeneous.

4.4. Application with brain tumors

One tumor dataset was affected by strong motion artefacts and in one dataset the tumor was close to the edge of the field of view. The remaining 12 datasets from the tumor patients were of high quality. Three have been selected due to interesting insights offered by SWI (Fig. 12); all are shown in Supplementary Information, Section H, Figures H-1 to H-13. In this section, Standard SWI denotes images generated by applying the Standard SWI pipeline to simulated single-echo data from the CLEAR-SWI multi-echo acquisition (Subsection 3.5).

In Patient A (Fig. 12, top row), who was diagnosed with a glioblastoma (WHO grade IV), multiple homodyne filtering artefacts were apparent in the proximity of the lesion (red arrows) in Standard SWI that were not present in CLEAR-SWI. The lesion shape was similar in CLEAR-SWI and the T_2* map, other than that the T_2* maps also contained a region of lower values, posterior to the tumor, which were mirrored only in the Standard SWI. We postulate that these are non-local effects from

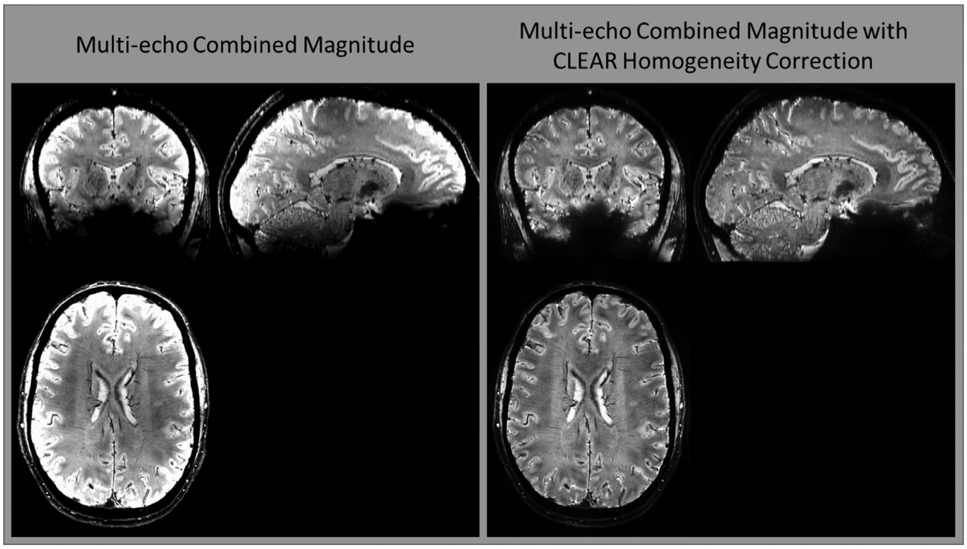

Fig. 8. Magnitude homogeneity correction. Multi-echo magnitude images combined with CNR weighting (left) show signal variation close to coil elements, which is effectively removed by the homogeneity correction (right). Note that the images are scaled to give similar contrast between the ventricles and neighboring white matter.

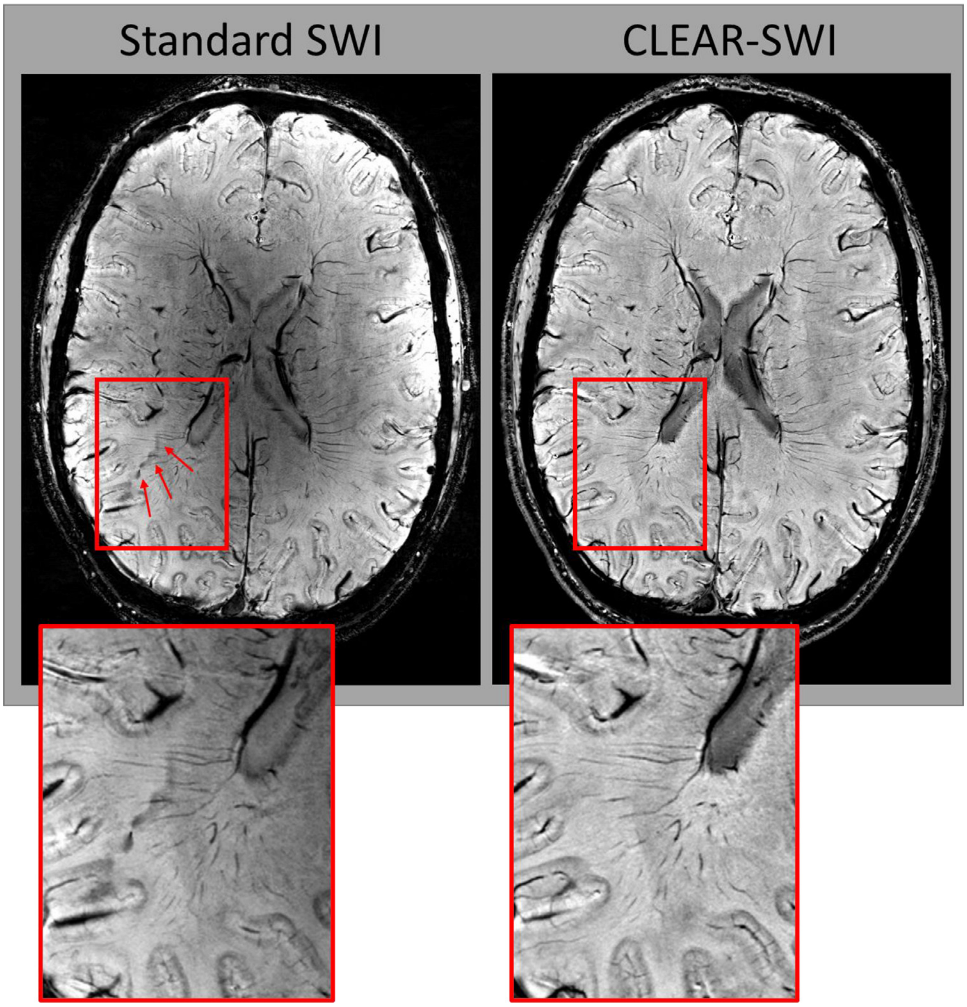

Fig. 9. Comparison of Standard SWI (*NE1*) and CLEAR-SWI (*NE6*; SNR-weighted softplus). The visibility of the veins is higher in CLEAR-SWI (red box). Standard SWI contains a "wormhole" artefact (red arrows) and is affected by strong intensity variations (being bright close to the surface of the brain and darker in central areas), while CLEAR-SWI is homogenous. Note that there are slight discrepancies in slice position, as the images are from different acquisitions.

strong field gradients emanating from the tumor, and that the CLEAR-SWI more accurately reflects local tissue composition.

Patient B (Fig. 12, middle row) was diagnosed with an anaplastic astrocytoma (WHO grade III). The vascularity directly surrounding the lesion could be seen in CLEAR-SWI, while Standard SWI overestimated the size of the lesion and obscured nearby veins (red arrow points to a hard-to-spot wrap artefact). Additionally, the clarity and distinction of veins in the whole image was visibly improved in CLEAR-SWI compared to Standard SWI. The parameter maps that were fitted from the CLEAR-SWI data (black arrows) illustrate the extent and state of the abnormal tissue, which appeared hyperintense on the $T_2{}^*$ map and hypointense in the central part containing necrosis. The T_1-weighted M_0 image pro-

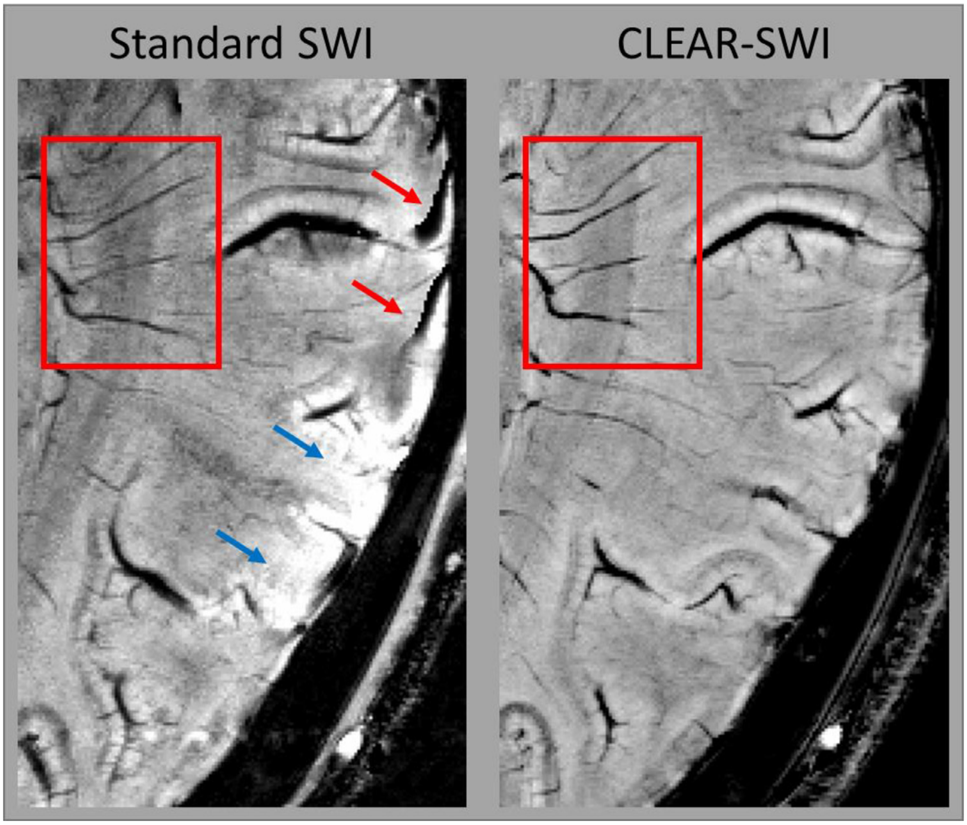

Fig. 10. Comparison of artefacts and vein visibility in Standard SWI (*NE1*) and CLEAR-SWI (*NE6*; SNR-weighted). Standard SWI (left) shows homodyne artefacts (red arrows) and inhomogeneity close to a coil (blue arrows); artefacts not present in CLEAR-SWI. Veins are more clearly resolved in CLEAR-SWI (red box). Note that there are slight discrepancies in slice position as the images are from different acquisitions.

vided unambiguous identification of CSF, which was hypointense, and some information about T_1 changes in abnormal tissue. It was, however, more affected by Gibbs ringing than the other contrast images.

The bottom row of Fig. 12 shows Patient C, who was diagnosed with an intra-axial mass lesion from a metastasized adenocarcinoma. In Standard SWI, strong wrap artefacts as well intensity variations led to poor image quality, while CLEAR-SWI allowed the vascularity inside the tumor to be visualized without artefacts.

Summarizing the tumor data, Standard SWI suffered from homodyne wrap artefacts in the region of the tumor in 10 out of 13 patients. These were particularly prominent in mIPs (see Supplementary Information, Appendix H). CLEAR-SWI was not affected by artefacts and provided high quality imaging of the veins, necrotic tissue (iron deposits) and contrast at the boundary of the central tumor region. There were elevated T_2^* values inside the larger region affected by the tumor and the T_1-weighted CLEAR M_0 showed contrast changes which were more localized to the immediate tumor region and, in some cases, contrast between an extended region around the tumor and normal appearing tissue (see Sup. Figs. H-4, H-8, H-9, H-13).

To compare Standard SWI and CLEAR-SWI in a clinical setting, a neuroradiologist (ST), a neurosurgeon (GW) and a neurologist (CE) were asked to rate the 13 tumor cases according to four criteria. The results in Fig. 13 show a significant improvement of CLEAR-SWI compared to Standard SWI in all four criteria. Especially the presence of artefacts was rated high in Standard SWI and very low in CLEAR-SWI.

5. Discussion

We have presented a comprehensive improvement to SWI acquisition and processing – Contrast-weighted, Laplace-unwrapped, bipolar multi-Echo, ASPIRE-combined, homogeneous, improved Resolution SWI (CLEAR-SWI) – which addresses the shortcomings of Standard SWI methods at high and ultra-high field. Employing bipolar multi-echo sequences with ASPIRE phase combination, Laplacian unwrapping and magnitude homogeneity correction avoids wrap-like artefacts and reduces signal loss in regions of high ΔB_0 gradients and intensity variations due to inhomogeneous B_1. Additional SNR and CNR improvements were achieved with weighted echo combination and sigmoidal phase masking functions. The phase processing in the first steps of CLEAR-SWI allows the phase to be used for the calculation of QSMs, in contrast to the homodyne filtering approach used in Standard SWI. In clinical application, CLEAR-SWI eliminated artefacts which could be interpreted as microbleeds, provided improved SNR and offered benefits associated with multi-echo acquisition, such as high resolution M_0 and T_2^* mapping.

5.1. Coil Combination

The fast and robust online coil combination for multi-echo acquisitions, ASPIRE (Eckstein et al., 2018a), generates combined phase images with no phase singularities and optimal CNR, independent of field strength and coil configuration. Other commonly applied phase combination schemes reduce CNR (phase difference in GEPCI; Luo et al., 2012), are prone to wrap-like artefacts (homodyne filter and combination; Denk and Rauscher, 2010), require a reference coil (Roemer/Sense; Pruessmann et al., 1999; Roemer et al., 1990), do not perform well at high field strengths (adaptive combine; Walsh et al., 2000) or are currently not robust enough for online application (VRC; Parker et al., 2014). Additional benefits of ASPIRE are that all non-B_0-related sources of phase are removed, so phase images are scaled, wrapped fieldmaps, and no high-pass filtering is applied, meaning that phase images can be used – after unwrapping – for QSM. MCPC-3D-S (Eckstein et al., 2018a), being closely related to and sharing most of the benefits of ASPIRE, would be another option for coil combination; it is not subject to the echo times restrictions of ASPIRE, but requires unwrapping of one 3D volume.

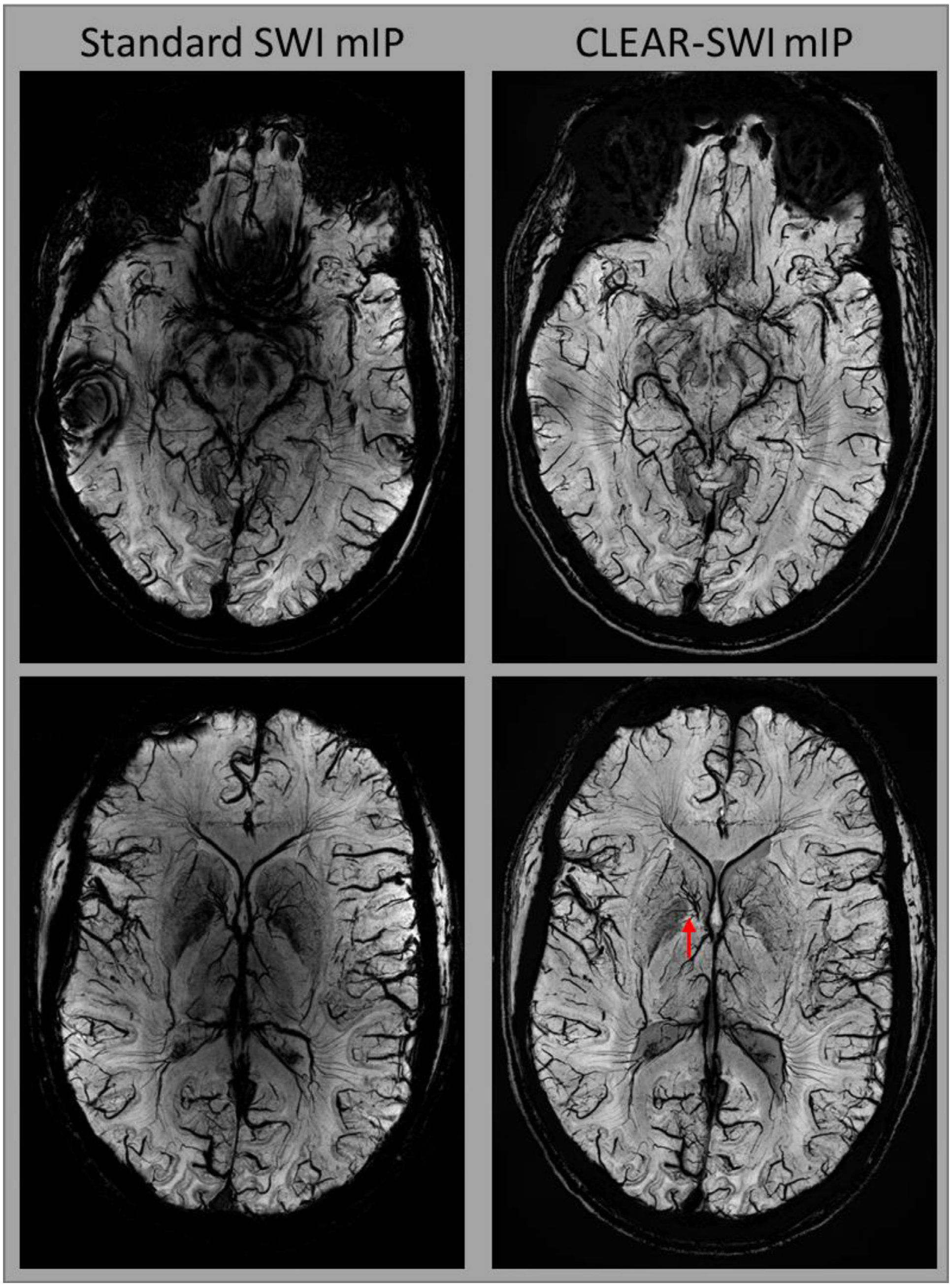

Fig. 11. Comparison between mIPs of Standard single-echo SWI (*NE1*) and multi-echo CLEAR-SWI (*NE6*; SNR-weighted softplus) in slices at the level of the substantia nigra (top) and striatum (bottom). Standard SWI has marked signal dropouts and strong wrap-like artefacts in lower slices (top left). Fine structures are more clearly visible in CLEAR-SWI (red arrow). Standard SWI shows large intensity variations over the image which are not present in CLEAR-SWI. In CLEAR-SWI, a very small vein can be seen inside the globus pallidus (directly above red arrow), which is unrecognizable in Standard SWI.

5.2. *Separate multi-echo combination of phase and magnitude*

In contrast to prior approaches in which an SWI is calculated from each echo and averaged (Denk and Rauscher, 2010), in which unresolved wrap artefacts in each SWI accumulate in the combined image, or artefact-affected parts of SWIs from later echoes are replaced by images from earlier ones (Oh et al., 2014), in CLEAR-SWI, magnitude and phase are combined over echoes, with dedicated weighting factors, prior to SWI calculation. This makes it possible to exploit the specific noise characteristics of phase and magnitude individually to increase the SNR/CNR in SWI.

5.3. *Magnitude multi-echo combination*

The multi-echo combination of the magnitude we outline offers a range of different contrast possibilities, which can be chosen for the specific use case. Magnitude images can be combined to optimize SNR using RSS combination over echoes (Jutras et al., 2017), which minimizes signal dropouts in areas of high B_0 variation. This might be the contrast best suited to imaging veins close to the skull base (Barrett et al., 2017). Alternatively, CNR between specific tissues can be optimized on the basis of literature values for relaxation times, or by calculating the optimal weights from ROIs drawn in the tissues. Although CNR-weighting provides optimal contrast in theory, two effects should be taken into account; an often spatially varying flip angle distribution and a possible crossover point, in which case some echoes might receive a negative weighting. For the magnitude averaging as performed by Brainovich et al. (2009), a lower SNR was found but the CNR values were comparable to the other presented multi-echo combinations. An additional softplus scaling step applied to the SNR-weighted combined magnitude produced a contrast which was similar to single-echo SWI, but in which images retained the advantages of the proposed magnitude combination - increased SNR and reduced signal dropouts.

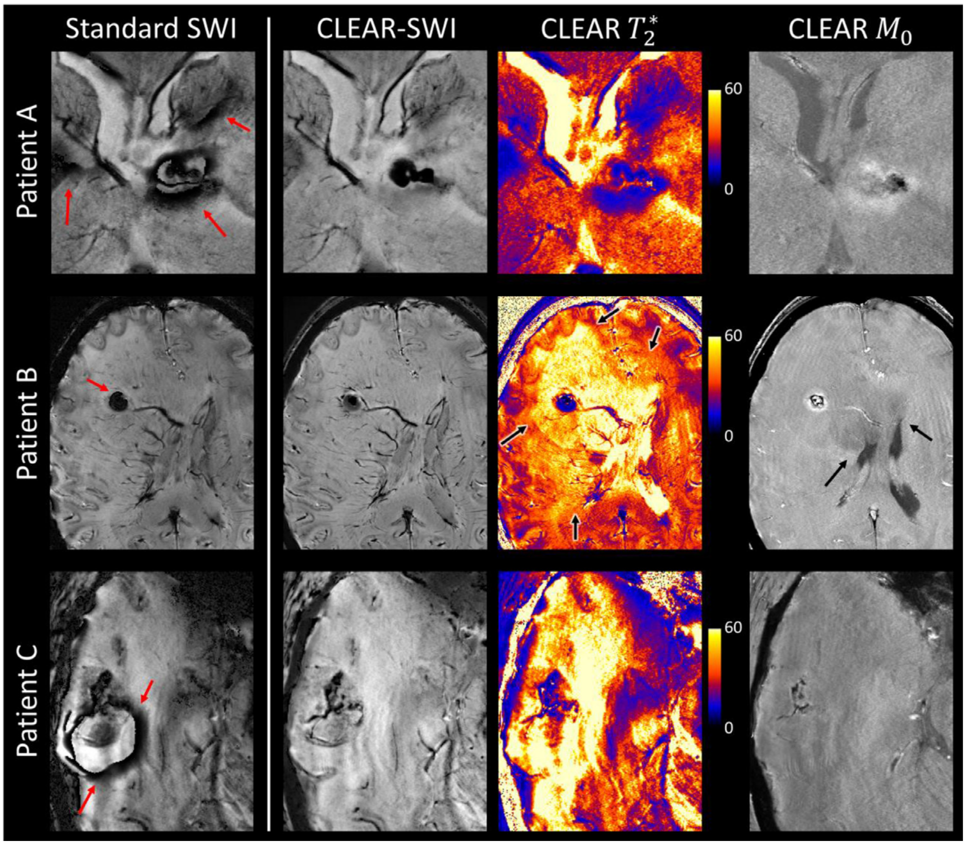

Fig. 12. Comparison of Standard SWI with WM-GM CNR-weighted CLEAR-SWI in three patients with brain tumors. Patient A) glioblastoma IV: the lesion leads to strong phase artefacts in Standard SWI (red arrows point to wrap artefacts). The shape of the lesion in CLEAR-SWI corresponds to the shape seen in T_2*, while T_2* also presents hypointense values below the necrosis that are not strictly tissue related, but arise from the strong field gradient. Patient B) anaplastic astrocytoma III: Standard SWI overestimates the size of the lesion due to a phase wrap artefact (red arrow). Patient C) metastasis – adenocarcinoma: Standard SWI suffers from severe phase wrap artefacts (red arrows) and inhomogeneous magnitude, which are both resolved in CLEAR-SWI. For all three patients, the clarity of veins and structures is visibly higher in CLEAR-SWI than in Standard SWI. The T_2* map and T_1-weighted M_0 provide additional contrast (black arrows). Note that all images are single slices and not mIPs.

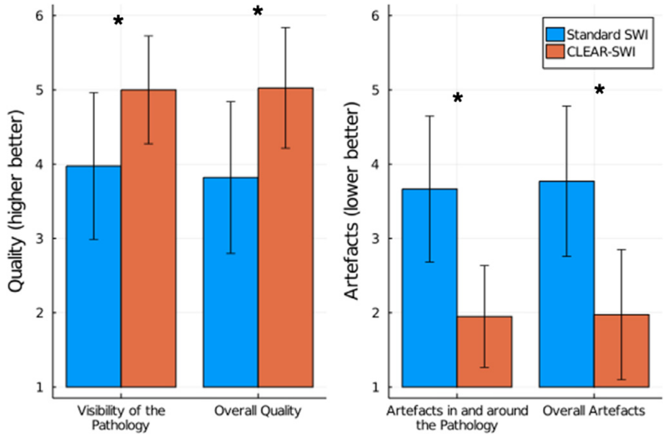

Fig. 13. Expert rating of Standard SWI and CLEAR-SWI of 13 Tumor cases according to four criteria. Each criterium was rated significantly better ($p < 0.01$) in CLEAR-SWI than in Standard SWI (Wilcoxon signed rank test).

5.4. *Phase multi-echo combination*

CLEAR-SWI integrates the phase information from all echoes using inverse-variance-weighted combination of the Laplacian-unwrapped and high-pass filtered phase. This maximizes the SNR of the combined phase in a multi-echo acquisition, similar to the weighted B_0 frequency combination (Quinn et al., 2014). The calculation of B_0 entails computationally demanding and error-prone quantitative (region-growing or path-based) unwrapping, however, which is avoided if a Laplacian unwrapping method is used. The mono-exponential fitting approach taken by Luo et al. (2012) is inferior in terms of SNR and also too computationally demanding to be executed online on the image reconstruction hardware, which was an important aspect of our study.

5.5. *Phase mask generation*

In Standard SWI, the magnitude is multiplied a number of times by a phase mask which is a linear function of phase values, resulting in a function of the degree of multiplications (Haacke et al., 2004; Reichenbach et al., 1997). The phase mask we propose in CLEAR-SWI uses an adaptive sigmoid function (tanh), which leads to improved visibility of small structures, e.g. veins and iron rings. A similar approach was taken by Quinn et al. (2014), who used a piecewise-defined cosine function. Tanh has the advantage that it is defined over the entire phase range and provides some contrast for negative phase values; a more gradual scaling than the abrupt setting of all negative phase values to 1, lending SWIs a more natural appearance.

5.6. *Phase unwrapping and high-pass filtering*

The use of Laplacian phase unwrapping (Schofield and Zhu, 2003) in CLEAR-SWI avoids the homodyne wrap-like artefacts which adversely affect SWI (Li et al., 2015), especially at high field strengths. Rauscher et al. (2003) have previously proposed region-growing unwrapping to achieve the same end. Whilst undoubtedly less prone to residual wraps than homodyne filtering, region-growing methods can have lengthy processing times and be prone to leaving or propagating wraps through the volume in highly wrapped regions with low SNR, leading Oh et al. (2014) to propose Laplacian unwrapping as an alternative. As we have shown here that this provides effective unwrapping even in the inferior brain, close to bone-air interfaces - regions which have seen interest in integrating SWI information into endoscopic endonasal surgical planning (Barrett et al., 2017; Rutland et al., 2020). Additional background field removal (Bollmann et al., 2019; Liu et al.,

2011; Schweser et al., 2017; Wu et al., 2012b) could allow a larger kernel size for high-pass filtering, which would enable phase weighting of larger structures, although care has to be taken when applying these methods to images containing pathologies.

5.7. Magnitude homogeneity correction

The homogeneity correction of the magnitude we propose here facilitates the visual assessment of ultra-high field SWI by reducing the need to repeatedly window regions under examination. Alternative methods we tested were found either not to be robust, to require manual intervention or be computationally demanding (Damen and Cai, 2018; Sled et al., 1998; Vovk et al., 2007) (results not shown). The fast and robust homogeneity correction approach we developed for CLEAR-SWI (Eckstein et al., 2018b) removed the strong bias field at 3 T and 7 T, and was not compromised by the presence of large hypo- or hyper-intense brain tumors. This is primarily a benefit for ultra-high field systems, which do not have the possibility to correct for coil sensitivities using a homogeneous reference coil image.

5.8. Comparison with advanced SWI methods

The method of Oh et al. (2014) uses a multi-echo acquisition to achieve a similar central objective as CLEAR-SWI – the avoidance of homodyne artefacts – but is essentially calculating a single-echo SWI (from the second echo) and replacing corrupted parts with an SWI from a fitted magnitude and first echo phase. It can therefore not benefit from the improved SNR/CNR available from multi-echo acquisitions and is very limited in the choice of its echo times. The two SWI methods in the literature that are most similar to CLEAR-SWI are GEPCI (Luo et al., 2012) and the multi-echo SWI described by Quinn et al. (2014). Like CLEAR-SWI, GEPCI utilizes a multi-echo sequence to produce multiple output images (SWI, T_2*, M_0, and newly devised contrasts). The method of Quinn et al. focusses primarily on phase processing, improving the echo combination of the phase and introducing a nonlinear phase masking function (Quinn et al., 2014). Compared to GEPCI and Quinn et al., CLEAR-SWI yielded equal or better CNR in the magnitude used for SWI, better visibility of veins and avoided inhomogeneities. Quinn et al. was conceived for lower field strengths and produced more artefacts than Standard SWI, making it unsuitable for application at 7 T. The voxelwise fitting in GEPCI was computationally highly demanding, limiting its application to offline processing.

Other methods propose the use of flow compensated sequences for SWI at 3 T (Du and Jin, 2008; Wu et al., 2016) to reduce the effect of arteries appearing as false veins, however, this entails additional gradients between the acquisition of echoes, lowering the duty cycle and therefore leading to reduced SNR. Because of this, and the lack of fully flow compensated sequences for clinical use, we decided to not explore flow compensation in this study. The CLEAR-SWI post-processing steps make no assumptions about the specific type of multi-echo sequence used, however, so we would not anticipate changes in the performance of CLEAR-SWI when applied to flow-compensated data.

Especially if applied at ultra-high field, coil combination is a crucial step to obtain artefact-free phase and if the echo time restrictions of ASPIRE cannot be fulfilled due to additional flow compensation gradients, MCPC-3D-S (Eckstein et al., 2018a) could be used as an alternative, which however, requires one additional unwrapping step.

5.9. Novel and published constituents of CLEAR-SWI

In addition to introducing novel and improved processing steps, CLEAR-SWI makes use of effective methods from the literature. This includes ASPIRE coil combination, Laplacian unwrapping for SWI, SNR-weighted echo combination for magnitude images and inverse-variance-weighted echo combination for phase images. Novel elements of CLEAR-SWI are the CNR-weighted echo combination and homogeneity correction (Eckstein et al., 2019). The sigmoidal phase masking is an improvement of the closely-related masking described by Quinn et al. (2014).

Some CLEAR-SWI steps could also benefit other imaging methods. The SNR- and CNR-weighted magnitude combination, for instance, could be applied in recombined multi-echo gradient-echo sequences (MERGE/MEDIC/MFFE) (Mekle et al., 2003; Nitz, 2002). The homogeneity correction was developed for SWI but has been shown to be effective with MPRAGE (Eckstein et al., 2019; Mugler and Brookeman, 1990) and FLAIR (unpublished). The non-linear magnitude scaling can increase the contrast of any magnitude image that is homogeneity corrected without affecting the local CNR. The use of ASPIRE (Eckstein et al., 2018a) for coil combination allows the phase data to be branched off for parallel Quantitative Susceptibility Mapping after that step, while the further phase processing (unwrapping and high-pass filtering) is quite specific to SWI. After application of the sigmoidal scaling, the phase mask itself is a high-quality image with very good clarity of veins and might be usable for direct visual inspection or further processing.

5.10. Computational efficiency

The processing steps in CLEAR-SWI are computationally efficient and can be calculated in less than 2 min on a high-resolution brain scan of 8 min acquisition time (*NE6*), which makes the method suitable for online implementation. The voxelwise fitting of T_2^* and M_0 to the multi-echo data is computationally expensive, however. In this study, T_2^* calculation was performed online using the fast numeric method NumART_2^* (Hagberg et al., 2002), which required less than 10 s on the same dataset.

5.11. Unwrapping, echo combination and QSM

Phase images generated with ASPIRE can be used for both SWI and QSM. The Laplacian unwrapping used in the CLEAR-SWI scheme is fast and robust but introduces an arbitrary background phase to each echo which requires that phase be high pass filtered prior to combination. We note that for QSM, which entails calculation of local B_0 (requiring unfiltered data), an alternative approach to echo combination is needed. As a first step, B_0 can be estimated from a non-linear fit to the complex multi-echo data, from which local B_0 can be calculated using Laplacian based methods (Biondetti et al., 2017, 2016). An alternative and potentially faster approach for data containing no phase offsets would be to spatially unwrap with a fast region-growing (Karsa and Shmueli, 2019) or path-following approach (Dymerska et al., 2021) and scale phase to generate B_0 maps which could be combined using weighted averaging. The resulting combined B_0 map could be used for both SWI and QSM. In the current scheme, SWI would be generated sufficiently quickly to be available online for reporting, and wrapped phase data would be exported offline for processing with a dedicated QSM pipeline. Recent methods propose replacing the phase-based contrast in SWI with susceptibility-based contrast by first calculating QSM (Gho et al., 2014; Liu et al., 2014), which is less orientation dependent and has a local, physical basis. Current disadvantages are, however, an often more noisy result, the removal of values close to the surface and difficulties in stable operation without expert user intervention. The added complexity is computationally demanding as well, involving additional precise masking, background field removal and dipole inversion.

5.12. Echo time constraints for ASPIRE and NumART_2*

The approach outlined here entails constraints on the echo times. Bipolar ASPIRE requires that $T_{E,2} = 2\ T_{E,1}$ and $T_{E,3} = 3\ T_{E,1}$ – i.e. in most settings, equispaced echoes with echo spacing equal to the first echo time. Satisfying these requirements can incur an SNR penalty, but does not do so if settings with optimum SNR are selected, according to Fig. 4. Consistent with this is the requirement of the fast T_2* fitting

method NumART_2^* (Hagberg et al., 2002), which needs all echoes to be equally spaced.

5.13. Clinical application in tumor imaging

No single-echo data was acquired for tumor patients in this study, but we demonstrate that simulated single-echo data can be generated from multi-echo data, and that these were good approximations to true single-echo acquisitions. In this study, simulated single-echo data allowed a reliable comparison between Standard SWI and CLEAR-SWI. Using the simulated single-echo magnitude in CLEAR-SWI might also be useful if identical contrast to Standard single-echo SWI is required, e.g. to allow comparison with prior imaging results or a transition from single-echo to multi-echo acquisitions in longitudinal studies.

Knowledge of the vascular environment in and around tumors can assist in assessing tumor type and grade (Grabner et al., 2017; Maeda et al., 1993; Wang et al., 2014). The complex structure of veins, oedema and necrotic tissue were well visualized using CLEAR-SWI; there were no artefacts despite strong phase variations close to pathologies and improved signal in tumors despite short T_2*. In Standard SWI, in contrast, there were phase artefacts within tumors and poor signal within some tumors, particularly in mIPs. The T_2* map which is available with CLEAR-SWI, provides information about the extent and the state of the abnormal tissue. The M_0 image provides some information about proton density and T_1, clearly showing CSF and some variation in abnormal tissue, although there is less contrast than in FLAIR images. In certain cases, what appears to be the boundary of the tumor was clearly visualized only in M_0, which was relatively insensitive to tissue changes in the immediately vicinity of the tumor.

The visibility of small veins was considerably improved in CLEAR-SWI compared with Standard SWI which may prove relevant in the study of other diseases with a vascular component, such as arteriovenous malformation (Miyasaka et al., 2012), stroke (Santhosh et al., 2009), and Sturge-Weber Syndrome (Hu et al., 2008).

5.14. Application at lower field strengths (3 T)

In many SWI approaches, the most fragile and field strength dependent parts are coil combination and phase filtering. Coil combination in CLEAR-SWI is performed using ASPIRE, which has been successfully applied at field strengths up to 9.4 T, and phase filtering is performed after unwrapping to avoid field strength dependent "wrap" artefacts. As B_0 and B_1 fields are less inhomogeneous at low field, the application of CLEAR-SWI at 3 T is unproblematic and our initial results have shown advantages over Standard SWI (not presented). Since some vendors already provide good homogeneity correction for 3 T, this step might not be required.

5.15. Spatial shift due to ΔB_0

CLEAR-SWI uses bipolar acquisitions, in which odd and even echoes are subject to equal and opposite susceptibility-related distortions. This can be calculated and corrected using a fieldmap calculated from the multi-echo phase data (Eckstein et al., 2018b). However, the shift is only relevant for very low bandwidths and because the bandwidths used here were rather high, the spatial shift was not corrected, being in the subvoxel range.

5.16. Application of CLEAR-SWI processing to single-echo SWI

The CLEAR-SWI magnitude processing steps of homogeneity correction and non-linear scaling, and the phase processing steps of unwrapping, high-pass filtering, and sigmoidal masking can also be applied to single-echo acquisitions. A comparison of Standard SWI with CLEAR-SWI steps applied to single-echo data is presented in Supplementary Information, Appendix F. The coil combination of phase images in the single-echo case is more problematic, however. Possible solutions are to estimate phase offsets using COMPOSER, which requires a short TE pre-scan, or ASPIRE, using a dual-echo prescan (Eckstein et al., 2018a; Robinson et al., 2011, 2017b), or to use the reference-free method VRC (Parker et al., 2014), which yields combined phase images without artefacts for brain imaging in most cases.

6. Conclusions

The improved SWI acquisition and processing steps we have presented in CLEAR-SWI address the shortcomings of Standard SWI methods at ultra-high field. In a comparison of CLEAR-SWI and Standard SWI measurements with the same acquisition time, the optimized combination of phase and magnitude data over RF coils and echoes in CLEAR-SWI led to higher contrast-to-noise images and a better resolution of small structures like veins, while Laplacian unwrapping avoided wrap-like artefacts observed in Standard SWI. The sigmoidal phase masking function reduced the noise introduced by conventional phase weighting, and intensity variations due to inhomogeneous B_1 were minimized with a magnitude homogeneity correction. In a comparison with other multi-echo SWI methods, CLEAR-SWI had equal or better visibility of veins and fewer artefacts. Significant improvements of CLEAR-SWI compared to Standard SWI were quantified for 13 tumor cases using expert ratings. All the applied methods are considered with a view towards online implementation on the imaging hardware in a fully automatic pipeline.

Declaration of Competing Interests

K.E. and S.R. are authors of a patent on the coil combination method ASPIRE ("Phase offset determination in magnetic resonance imaging", WO/2018/028855, PCT/EP2017/064131).

Acknowledgements

This study was funded by the Austrian Science Fund (FWF): 31452. Additional support was provided by Austrian Science Fund (FWF): KLI-646 and the Marie Skłodowska-Curie Action MS-fMRI-QSM 794298. The financial support by the Austrian Federal Ministry for Digital and Economic Affairs and the National Foundation for Research, Technology and Development is gratefully acknowledged. The funding institutions were not involved in carrying out this work or making decisions regarding it other than the initial approval of funding. This work was published in preliminary form (Eckstein et al., 2018b; Robinson et al., 2020).

Credit author statement

Korbinian Eckstein: Conceptualization, Methodology, Software, Validation, Visualization, Writing – Original Draft

Beata Bachrata: Investigation (SNR study), Data curation

Gilbert Hangel: Investigation (tumor measurements)

Georg Widhalm: Investigation (tumor patient recruitment), Validation

Christian Enzinger: Validation

Markus Barth: Conceptualization

Siegfried Trattnig: Funding acquisition, Validation

Simon Robinson: Conceptualization, Writing – Review & Editing, Supervision, Funding acquisition

Supplementary materials

Supplementary material associated with this article can be found, in the online version, at doi:10.1016/j.neuroimage.2021.118175.

References

Absinta, M., Sati, P., Schindler, M., Leibovitch, E.C., Ohayon, J., Wu, T., Meani, A., Filippi, M., Jacobson, S., Cortese, I.C.M., Reich, D.S., 2016. Persistent 7-tesla phase rim predicts poor outcome in new multiple sclerosis patient lesions. J. Clin. Invest. 126, 2597–2609. doi:10.1172/JCI86198.

Ashburner, J., Friston, K.J., 2005. Unified segmentation. Neuroimage 26, 839–851. doi:10.1016/j.neuroimage.2005.02.018.

Ayaz, M., Boikov, A.S., Haacke, E.M., Kido, D.K., Kirsch, W.M., 2010. Imaging cerebral microbleeds using susceptibility weighted imaging: One step toward detecting vascular dementia. J. Magn. Reson. Imaging 31, 142–148. doi:10.1002/jmri.22001.

Barrett, T.F., Dyvorne, H.A., Padormo, F., Pawha, P.S., Delman, B.N., Shrivastava, R.K., Balchandani, P., 2017. First application of 7T magnetic resonance imaging in endoscopic endonasal surgery of skull base tumors. World Neurosurg. 103, 600–610. doi:10.1016/j.wneu.2017.03.088.

Bernstein, M.A., King, K.F., Zhou, X.J., 2004. Handbook of MRI Pulse Sequences. Elsevier.

Bezanson, Jeff., Edelman, Alan., Karpinski, Stefan., Shah, V.B., 2017. Julia: a fresh approach to numerical computing.. SIAM Rev. 59, 65–98. doi:10.1137/141000671.

Bian, W., Hess, C.P., Chang, S.M., Nelson, S.J., Lupo, J.M., 2014. Susceptibility-weighted MR imaging of radiation therapy-induced cerebral microbleeds in patients with glioma: a comparison between 3T and 7T. Neuroradiology 56, 91–96. doi:10.1007/s00234-013-1297-8.

Biondetti, E., Karsa, A., Thomas, D.L., Shmueli, K., 2017. Evaluating The Accuracy of Susceptibility Maps Calculated from Single-Echo versus Multi-Echo Gradient-Echo Acquisitions, in: Proceedings of the 25th Annual Meeting ISMRM. Presented at the ISMRM, Honolulu, Hawaii, USA.

Biondetti, E., Thomas, D.L., Shmueli, K., 2016. Application of Laplacian-based Methods to Multi-echo Phase Data for Accurate Susceptibility Mapping, in: Proceedings of the 24th Annual Meeting ISMRM. Presented at the ISMRM, Singapore.

Bollmann, S., Kristensen, M.H., Larsen, M.S., Olsen, M.V., Pedersen, M.J., Østergaard, L.R., O'Brien, K., Langkammer, C., Fazlollahi, A., Barth, M., 2019. SHARQnet – Sophisticated harmonic artifact reduction in quantitative susceptibility mapping using a deep convolutional neural network. Zeitschrift für Medizinische Physik, Special Issue 29, 139–149. doi:10.1016/j.zemedi.2019.01.001.

Brainovich, V., Sabatini, U., Hagberg, G.E., 2009. Advantages of using multiple-echo image combination and asymmetric triangular phase masking in magnetic resonance venography at 3 T.. Magn Reson Imaging 27, 23–37. doi:10.1016/j.mri.2008.05.006.

Dal-Bianco, A., Hametner, S., Grabner, G., Schernthaner, M., Kronnerwetter, C., Reitner, A., Vass, C., Kircher, K., Auff, E., Leutmezer, F., Vass, K., Trattnig, S., 2015. Veins in plaques of multiple sclerosis patients – a longitudinal magnetic resonance imaging study at 7 Tesla –. Eur Radiol 25, 2913–2920. doi:10.1007/s00330-015-3719-y.

Damen, F.C., Cai, K., 2018. B1− non-uniformity correction of phased-array coils without measuring coil sensitivity. Magn. Reson. Imaging 51, 20–28. doi:10.1016/j.mri.2018.04.009.

Deistung, A., Rauscher, A., Sedlacik, J., Stadler, J., Witoszynskyj, S., Reichenbach, J.R., 2008. Susceptibility weighted imaging at ultra high magnetic field strengths: Theoretical considerations and experimental results. Magn. Reson. Med. 60, 1155–1168. doi:10.1002/mrm.21754.

Denk, C., Rauscher, A., 2010. Susceptibility weighted imaging with multiple echoes. J. Magn. Reson. Imaging 31, 185–191. doi:10.1002/jmri.21995.

Di Ieva, A., Göd, S., Grabner, G., Grizzi, F., Sherif, C., Matula, C., Tschabitscher, M., Trattnig, S., 2013. Three-dimensional susceptibility-weighted imaging at 7 T using fractal-based quantitative analysis to grade gliomas. Neuroradiology 55, 35–40. doi:10.1007/s00234-012-1081-1.

Du, Y.P., Jin, Z., 2008. Simultaneous acquisition of MR angiography and venography (MRAV). Magn. Reson. Med. 59, 954–958. doi:10.1002/mrm.21581.

Dymerska, B., Eckstein, K., Bachrata, B., Siow, B., Trattnig, S., Shmueli, K., Robinson, S.D., 2021. Phase unwrapping with a rapid opensource minimum spanning tree algorithm (ROMEO). Magn. Reson. Med. 85, 2294–2308. doi:10.1002/mrm.28563.

Eckstein, K., Bachrata, B., Robinson, S., 2021. CLEAR-SWI (MRI images). Harvard Dataverse. https://doi.org/10.7910/DVN/4ZUFTH

Eckstein, K., Dymerska, B., Bachrata, B., Bogner, W., Poljanc, K., Trattnig, S., Robinson, S.D., 2018a. Computationally efficient combination of multi-channel phase data from multi-echo acquisitions (ASPIRE). Magn. Reson. Med. 79, 2996–3006. https://doi.org/10.1002/mrm.26963

Eckstein, K., Trattnig, S., Robinson, S.D., 2019. A Simple homogeneity correction for neuroimaging at 7T, in: Proceedings of the 27th Annual Meeting ISMRM. Presented at the ISMRM, Montréal, Québec, Canada.

Eckstein, K., Trattnig, S., Robinson, S.D., 2018b. High resolution frequency difference mapping using ASPIRE phase combination, in: Proceedings of the 26th Annual Meeting ISMRM. Presented at the ISMRM, Paris, France.

Filippi, M., Brück, W., Chard, D., Fazekas, F., Geurts, J.J.G., Enzinger, C., Hametner, S., Kuhlmann, T., Preziosa, P., Rovira, À., Schmierer, K., Stadelmann, C., Rocca, M.A., 2019. Association between pathological and MRI findings in multiple sclerosis. Lancet Neurol. 18 (18), 198–210. doi:10.1016/S1474-4422, 30451-4.

Frischer, J.M., Göd, S., Gruber, A., Saringer, W., Grabner, G., Gatterbauer, B., Kitz, K., Holzer, S., Kronnerwetter, C., Hainfellner, J.A., Knosp, E., Trattnig, S., 2012. Susceptibility-weighted imaging at 7T: Improved diagnosis of cerebral cavernous malformations and associated developmental venous anomalies. NeuroImage 1, 116–120. doi:10.1016/j.nicl.2012.09.005.

Gho, S.-M., Liu, C., Li, W., Jang, U., Kim, E.Y., Hwang, D., Kim, D.-H., 2014. Susceptibility map-weighted imaging (SMWI) for neuroimaging. Magn. Reson. Med. 72, 337–346. doi:10.1002/mrm.24920.

Gilbert, G., Savard, G., Bard, C., Beaudoin, G., 2012. Quantitative comparison between a multiecho sequence and a single-echo sequence for susceptibility-weighted phase imaging.. Magnet. Resonance Imaging 30, 722–730. doi:10.1016/j.mri.2012.02.008.

Goerner, F.L., Clarke, G.D., 2011. Measuring signal-to-noise ratio in partially parallel imaging MRI. Med. Phys. 38, 5049–5057. doi:10.1118/1.3618730.

Grabner, G., Kiesel, B., Wöhrer, A., Millesi, M., Wurzer, A., Göd, S., Mallouhi, A., Knosp, E., Marosi, C., Trattnig, S., Wolfsberger, S., Preusser, M., Widhalm, G., 2017. Local image variance of 7 Tesla SWI is a new technique for preoperative characterization of diffusely infiltrating gliomas: correlation with tumour grade and IDH1 mutational status. Eur Radiol. 27, 1556–1567. doi:10.1007/s00330-016-4451-y.

Haacke, E., Reichenbach, J.R., 2011. Susceptibility Weighted Imaging in MRI: Basic Concepts and Clinical Applications. Wiley-Blackwell.

Haacke, E.M., Xu, Y., Cheng, Y.-C.N., Reichenbach, J.R., 2004. Susceptibility weighted imaging (SWI). Magn. Reson. Med. 52, 612–618. https://doi.org/10.1002/mrm.20198

Hagberg, G.E., Indovina, I., Sanes, J.N., Posse, S., 2002. Real-time quantification of T2* changes using multiecho planar imaging and numerical methods. Magn. Reson. Med. 48, 877–882. doi:10.1002/mrm.10283.

Hu, J., Yu, Y., Juhasz, C., Kou, Z., Xuan, Y., Latif, Z., Kudo, K., Chugani, H.T., Haacke, E.M., 2008. MR susceptibility weighted imaging (SWI) complements conventional contrast enhanced T1 weighted MRI in characterizing brain abnormalities of Sturge-Weber Syndrome. J. Magn. Reson. Imaging 28, 300–307. doi:10.1002/jmri.21435.

Jenkinson, M., Bannister, P., Brady, M., Smith, S., 2002. Improved optimization for the robust and accurate linear registration and motion correction of brain images. Neuroimage 17, 825–841. doi:10.1016/s1053-8119(02)91132-8.

Jutras, J.-D., Wachowicz, K., Gilbert, G., De Zanche, N., 2017. SNR efficiency of combined bipolar gradient echoes: comparison of three-dimensional FLASH, MPRAGE, and multiparameter mapping with VFA-FLASH and MP2RAGE. Magn. Reson. Med. 77, 2186–2202. doi:10.1002/mrm.26306.

Karsa, A., Shmueli, K., 2019. SEGUE: a speedy rEgion-growing algorithm for unwrapping estimated phase. IEEE Trans. Med. Imaging 38, 1347–1357. doi:10.1109/TMI.2018.2884093.

Koopmans, P.J., Manniesing, R., Niessen, W.J., Viergever, M.A., Barth, M., 2008. MR venography of the human brain using susceptibility weighted imaging at very high field strength. MAGMA 21, 149–158. doi:10.1007/s10334-007-0101-3.

Li, N., Wang, W.-T., Pham, D.L., Butman, J.A., 2015. Artifactual microhemorrhage generated by susceptibility weighted image processing. J. Magn. Reson. Imaging 41, 1695–1700. doi:10.1002/jmri.24728.

Liu, S., Buch, S., Chen, Y., Choi, H.-S., Dai, Y., Habib, C., Hu, J., Jung, J.-Y., Luo, Y., Utriainen, D., Wang, M., Wu, D., Xia, S., Haacke, E.M., 2017. Susceptibility-weighted imaging: current status and future directions. NMR Biomed. 30, e3552. doi:10.1002/nbm.3552.

Liu, S., Mok, K., Neelavalli, J., Cheng, Y.-C.N., Tang, J., Ye, Y., Haacke, E.M., 2014. Improved MR venography using quantitative susceptibility-weighted imaging. J. Magn. Reson. Imaging 40, 698–708. doi:10.1002/jmri.24413.

Liu, T., Khalidov, I., Rochefort, L.de, Spincemaille, P., Liu, J., Tsiouris, A.J., Wang, Y., 2011. A novel background field removal method for MRI using projection onto dipole fields (PDF). NMR Biomed. 24, 1129–1136. doi:10.1002/nbm.1670.

Luo, J., Jagadeesan, B.D., Cross, A.H., Yablonskiy, D.A., 2012. Gradient Echo Plural Contrast Imaging — Signal model and derived contrasts: T2*, T1, Phase, SWI, T1f, FST2*and T2*-SWI. NeuroImage 60, 1073–1082. https://doi.org/10.1016/j.neuroimage.2012.01.108

Maeda, M., Itoh, S., Kimura, H., Iwasaki, T., Hayashi, N., Yamamoto, K., Ishii, Y., Kubota, T., 1993. Tumor vascularity in the brain: evaluation with dynamic susceptibility-contrast MR imaging. Radiology 189, 233–238. doi:10.1148/radiology.189.1.8372199.

Mekle, R., Laine, A.F., Wu, E.X., 2003. Combined MR data acquisition of multicontrast images using variable acquisition parameters and K-space data sharing. IEEE Trans. Med. Imaging 22, 806–823. doi:10.1109/TMI.2003.815054.

Miyasaka, T., Taoka, T., Nakagawa, H., Wada, T., Takayama, K., Myochin, K., Sakamoto, M., Ochi, T., Akashi, T., Kichikawa, K., 2012. Application of susceptibility weighted imaging (SWI) for evaluation of draining veins of arteriovenous malformation: utility of magnitude images. Neuroradiology 54, 1221–1227. doi:10.1007/s00234-012-1029-5.

Moenninghoff, C., Kraff, O., Maderwald, S., Umutlu, L., Theysohn, J.M., Ringelstein, A., Wrede, K.H., Deuschl, C., Altmeppen, J., Ladd, M.E., Forsting, M., Quick, H.H., Schlamann, M., 2015. Diffuse axonal injury at ultra-high field MRI. PLoS One 10, e0122329. doi:10.1371/journal.pone.0122329.

Mugler, J.P., Brookeman, J.R., 1990. Three-dimensional magnetization-prepared rapid gradient-echo imaging (3D MP RAGE). Magn. Reson. Med. 15, 152–157. doi:10.1002/mrm.1910150117.

Najdenovska, E., Tuleasca, C., Jorge, J., Maeder, P., Marques, J.P., Roine, T., Gallichan, D., Thiran, J.-P., Levivier, M., Bach Cuadra, M., 2019. Comparison of MRI-based automated segmentation methods and functional neurosurgery targeting with direct visualization of the Ventro-intermediate thalamic nucleus at 7T. Sci. Rep. 9, 1119. doi:10.1038/s41598-018-37825-8.

Nitz, W.R., 2002. Fast and ultrafast non-echo-planar MR imaging techniques. Eur. Radiol. 12, 2866–2882. doi:10.1007/s00330-002-1428-9.

Noll, D.C., Nishimura, D.G., Macovski, A., 1991. Homodyne detection in magnetic resonance imaging. IEEE Trans. Med. Imaging 10, 154–163. doi:10.1109/42.79473.

Oh, S.S., Oh, S.-H., Nam, Y., Han, D., Stafford, R.B., Hwang, J., Kim, D.-H., Park, H., Lee, J., 2014. An improved susceptibility weighted imaging method using multi-echo acquisition. Magn. Reson. Med 72, 452–458. doi:10.1002/mrm.24940.

Ong, B.C., Stuckey, S.L., 2010. Susceptibility weighted imaging: a pictorial review. J. Med. Imaging Radiation Oncol. 54, 435–449. doi:10.1111/j.1754-9485.2010.02208.x.

Parker, D.L., Payne, A., Todd, N., Hadley, J.R., 2014. Phase reconstruction from multiple coil data using a virtual reference coil. Magn. Reson. Med. 72, 563–569. doi:10.1002/mrm.24932.

Peters, A.M., Brookes, M.J., Hoogenraad, F.G., Gowland, P.A., Francis, S.T., Morris, P.G.,

Bowtell, R., 2007. T2* measurements in human brain at 1.5, 3 and 7 T. Magnetic Resonance Imaging. Proceedings of the International School on Magnetic Resonance and Brain Function 25, 748–753 https://doi.org/10.1016/j.mri.2007.02.014.

Pittau, F., Baud, M.O., Jorge, J., Xin, L., Grouiller, F., Iannotti, G.R., Seeck, M., Lazeyras, F., Vulliémoz, S., Vargas, M.I., 2018. MP2RAGE and susceptibility-weighted imaging in lesional epilepsy at 7T. J. Neuroimaging 28, 365–369. doi:10.1111/jon.12523.

Pruessmann, K.P., Weiger, M., Scheidegger, M.B., Boesiger, P., 1999. SENSE: sensitivity encoding for fast MRI. Magn. Reson. Med. 42, 952–962.

Quinn, M.P., Gati, J.S., Klassen, L.M., Lin, A.W., Bird, J.R., Leung, S.E., Menon, R.S., 2014. Comparison of multiecho postprocessing schemes for SWI with use of linear and nonlinear mask functions. AJNR Am. J. Neuroradiol 35, 38–44. doi:10.3174/ajnr.A3584.

Rauscher, A., Barth, M., Reichenbach, J.R., Stollberger, R., Moser, E., 2003. Automated unwrapping of MR phase images applied to BOLD MR-venography at 3 Tesla. J. Magn. Reson. Imaging 18, 175–180. doi:10.1002/jmri.10346.

Reichenbach, J.R., Barth, M., Haacke, E.M., Klarhöfer, M., Kaiser, W.A., Moser, E., 2000. High-resolution MR venography at 3.0 tesla. J. Comput. Assist. Tomogr. 24, 949–957. doi:10.1097/00004728-200011000-00023.

Reichenbach, J.R., Venkatesan, R., Schillinger, D.J., Kido, D.K., Haacke, E.M., 1997. Small vessels in the human brain: MR venography with deoxyhemoglobin as an intrinsic contrast agent. Radiology 204, 272–277. doi:10.1148/radiology.204.1.9205259.

Robinson, S., Grabner, G., Witoszynskyj, S., Trattnig, S., 2011. Combining phase images from multi-channel RF coils using 3D phase offset maps derived from a dual-echo scan. Magn. Reson. Med. 65, 1638–1648. doi:10.1002/mrm.22753.

Robinson, S.D., Bredies, K., Khabipova, D., Dymerska, B., Marques, J.P., Schweser, F., 2017a. An illustrated comparison of processing methods for MR phase imaging and QSM: combining array coil signals and phase unwrapping. NMR Biomed. 30. doi:10.1002/nbm.3601.

Robinson, S.D., Dymerska, B., Bogner, W., Barth, M., Zaric, O., Goluch, S., Grabner, G., Deligianni, X., Bieri, O., Trattnig, S., 2017b. Combining phase images from array coils using a short echo time reference scan (COMPOSER). Magn. Reson. Med. 77, 318–327. doi:10.1002/mrm.26093.

Robinson, S.D., Hangel, G., Bachrata, B., Ehrmann, A., Trattnig, S., Enzinger, C., Barth, M., Eckstein, K., 2020. Improved Susceptibility Weighted Imaging using bipolar multi-echo acquisition and optimized processing of phase and magnitude data: CLEAR-SWI. In: Proceedings of the 28th Annual Meeting ISMRM. Presented at the ISMRM, Virtual Conference.

Roemer, P.B., Edelstein, W.A., Hayes, C.E., Souza, S.P., Mueller, O.M., 1990. The NMR phased array. Magn. Reson. Med. 16, 192–225. doi:10.1002/mrm.1910160203.

Rovira, À., Wattjes, M.P., Tintoré, M., Tur, C., Yousry, T.A., Sormani, M.P., De Stefano, N., Filippi, M., Auger, C., Rocca, M.A., Barkhof, F., Fazekas, F., Kappos, L., Polman, C., Miller, D., Montalban, X.MAGNIMS study group, 2015. Evidence-based guidelines: MAGNIMS consensus guidelines on the use of MRI in multiple sclerosis-clinical implementation in the diagnostic process. Nat. Rev. Neurol. 11, 471–482. doi:10.1038/nrneurol.2015.106.

Rutland, J.W., Delman, B.N., Gill, C.M., Zhu, C., Shrivastava, R.K., Balchandani, P., 2020. Emerging use of ultra-high-field 7T MRI in the study of intracranial vascularity: state of the field and future directions. AJNR Am. J. Neuroradiol. 41, 2–9. doi:10.3174/ajnr.A6344.

Santhosh, K., Kesavadas, C., Thomas, B., Gupta, A.K., Thamburaj, K., Kapilamoorthy, T.R., 2009. Susceptibility weighted imaging: a new tool in magnetic resonance imaging of stroke. Clin. Radiol. 64, 74–83. doi:10.1016/j.crad.2008.04.022.

Schlamann, M., Maderwald, S., Becker, W., Kraff, O., Theysohn, J.M., Mueller, O., Sure, U., Wanke, I., Ladd, M.E., Forsting, M., Schaefer, L., Gizewski, E.R., 2010. Cerebral cavernous hemangiomas at 7 Tesla: initial experience. Acad. Radiol. 17, 3–6. doi:10.1016/j.acra.2009.10.001.

Schofield, M.A., Zhu, Y., 2003. Fast phase unwrapping algorithm for interferometric applications. Opt. Lett. 28, 1194–1196. doi:10.1364/OL.28.001194, OL.

Schweser, F., Deistung, A., Lehr, B.W., Reichenbach, J.R., 2011. Quantitative imaging of intrinsic magnetic tissue properties using MRI signal phase: an approach to in vivo brain iron metabolism? Neuroimage 54, 2789–2807. doi:10.1016/j.neuroimage.2010.10.070.

Schweser, F., Robinson, S., de Rochefort, L., Li, W., Bredies, K., 2017. An illustrated comparison of processing methods for phase MRI and QSM: Removal of background field contributions from sources outside the region of interest. NMR Biomed. 30. doi:10.1002/nbm.3604.

Sehgal, V., Delproposto, Z., Haacke, E.M., Tong, K.A., Wycliffe, N., Kido, D.K., Xu, Y., Neelavalli, J., Haddar, D., Reichenbach, J.R., 2005. Clinical applications of neuroimaging with susceptibility-weighted imaging. J. Magn. Reson. Imaging 22, 439–450. doi:10.1002/jmri.20404.

Sled, J.G., Zijdenbos, A.P., Evans, A.C., 1998. A nonparametric method for automatic correction of intensity nonuniformity in MRI data. IEEE Trans. Med. Imaging 17, 87–97. doi:10.1109/42.668698.

Sodickson, D.K., Griswold, M.A., Jakob, P.M., Edelman, R.R., Manning, W.J., 1999. Signal-to-noise ratio and signal-to-noise efficiency in SMASH imaging. Magn. Reson. Med. 41, 1009–1022. doi:10.1002/(SICI)1522-2594(199905)41:5<1009::AID-MRM21>3.0.CO;2-4.

Tan, I.L., van Schijndel, R.A., Pouwels, P.J., van Walderveen, M.A., Reichenbach, J.R., Manoliu, R.A., Barkhof, F., 2000. MR venography of multiple sclerosis. AJNR. Am. J. Neuroradiol. 21, 1039–1042.

Theysohn, J.M., Kraff, O., Maderwald, S., Barth, M., Ladd, S.C., Forsting, M., Ladd, M.E., Gizewski, E.R., 2011. 7 tesla MRI of microbleeds and white matter lesions as seen in vascular dementia. J. Magn. Reson. Imaging 33, 782–791. doi:10.1002/jmri.22513.

Vovk, U., Pernus, F., Likar, B., 2007. A review of methods for correction of intensity inhomogeneity in MRI. IEEE Trans. Med. Imaging 26, 405–421. doi:10.1109/TMI.2006.891486.

Walsh, D.O., Gmitro, A.F., Marcellin, M.W., 2000. Adaptive reconstruction of phased array MR imagery. Magn. Reson. Med. 43, 682–690 https://doi.org/10.1002/(sici)1522-2594(200005)43:5<682::aid-mrm10>3.0.co;2-g.

Wang, X., Zhang, H., Tan, Y., Qin, J., Wu, X., Wang, L., Zhang, L., 2014. Combined value of susceptibility-weighted and perfusion-weighted imaging in assessing WHO grade for brain astrocytomas. J. Magn. Reson. Imaging 39, 1569–1574. doi:10.1002/jmri.24312.

Wang, Y., Liu, T., 2015. Quantitative susceptibility mapping (QSM): Decoding MRI data for a tissue magnetic biomarker. Magn. Reson. Med. 73, 82–101. doi:10.1002/mrm.25358.

Wharton, S., Bowtell, R., 2013. Gradient echo based fiber orientation mapping using R2* and frequency difference measurements. Neuroimage 83, 1011–1023. doi:10.1016/j.neuroimage.2013.07.054.

Wharton, S., Bowtell, R., 2012. Fiber orientation-dependent white matter contrast in gradient echo MRI. Proc. Natl Acad. Sci. 109, 18559–18564. doi:10.1073/pnas.1211075109.

Wright, P.J., Mougin, O.E., Totman, J.J., Peters, A.M., Brookes, M.J., Coxon, R., Morris, P.E., Clemence, M., Francis, S.T., Bowtell, R.W., Gowland, P.A., 2008. Water proton T1 measurements in brain tissue at 7, 3, and 1.5 T using IR-EPI, IR-TSE, and MPRAGE: results and optimization. MAGMA 21, 121–130. doi:10.1007/s10334-008-0104-8.

Wu, B., Li, W., Avram, A.V., Gho, S.-M., Liu, C., 2012a. Fast and tissue-optimized mapping of magnetic susceptibility and T2* with multi-echo and multi-shot spirals.. NeuroImage, Neuroergonomics 59, 297–305. doi:10.1016/j.neuroimage.2011.07.019.

Wu, B., Li, W., Guidon, A., Liu, C., 2012b. Whole brain susceptibility mapping using compressed sensing. Magn. Reson. Med. 67, 137–147. doi:10.1002/mrm.23000.

Wu, D., Liu, S., Buch, S., Ye, Y., Dai, Y., Haacke, E.M., 2016. A fully flow-compensated multiecho susceptibility-weighted imaging sequence: the effects of acceleration and background field on flow compensation. Magn. Reson. Med. 76, 478–489. doi:10.1002/mrm.25878.
